# Differential metabolomic signatures in plasma and urine under mild and moderate hypothermia during cardiopulmonary bypass

**DOI:** 10.1038/s41598-025-24913-9

**Published:** 2025-11-20

**Authors:** Oguzhan Durmaz, Cemil Can Eylem, Evren Ozcinar, Emirhan Nemutlu, Osman Dag, Burak Derkus, Emel Emregul

**Affiliations:** 1 Department of Chemistry, Ankara University Graduate School of Natural and Applied Sciences, Ankara, Turkey; 2 Department of Analytical Chemistry, Hacettepe University Faculty of Pharmacy, Ankara, Turkey; 3 Department of Cardiovascular Surgery, Ankara University School of Medicine, Ankara, Turkey; 4 Department of Biostatistics, Hacettepe University Faculty of Medicine, Ankara, Turkey; 5 Department of Chemistry, Division of Biochemistry, Faculty of Science, Ankara University, Ankara, Turkey

**Keywords:** Metabolomics, Cardiopulmonary bypass, Hypothermia, Plasma, Urine, Biomarkers, Metabolomics, Biochemistry, Chemical biology, Metabolomics, Biomarkers, Predictive markers

## Abstract

While the organ-protective effects of hypothermia during cardiopulmonary bypass (CPB) are well known, the metabolomic impacts of different hypothermia levels remain unclear. This study aimed to evaluate the effects of mild (32–35 °C, *n* = 15) and moderate (26–31 °C, *n* = 14) hypothermia on plasma and urinary metabolomic profiles in adults undergoing CPB. Untargeted metabolomic analysis was conducted using gas chromatography–mass spectrometry (GC-MS) and liquid chromatography–quadrupole time-of-flight mass spectrometry (LC-qTOF-MS), which identified 137 plasma and 101 urinary metabolites. Statistical and multivariate analyses revealed significant differences in metabolic profiles between the two groups. In plasma, reduced levels of citric acid, proline, and inosine were observed in the mild hypothermia group, whereas myristic acid and glyceraldehyde levels were lower in the moderate group. In urine, the mild hypothermia group showed increased levels of creatinine and 5,6-dihydroxyeicosatrienoic acid (5,6-DHET), along with decreased levels of 2-hydroxybutyric acid, N-acetylserine, and oxaloacetic acid, indicating reduced renal stress. Conversely, elevated levels of C17-sphinganine and ceramides in the moderate group suggested altered lipid metabolism and greater cellular stress. Moderate hypothermia was associated with higher metabolic stress, whereas mild hypothermia was associated with relative metabolic stability. These findings present candidate biomarkers for optimizing hypothermia strategies during CPB.

**Supplementary Information:**

The online version contains supplementary material available at 10.1038/s41598-025-24913-9.

## Introduction

Cardiopulmonary bypass (CPB) allows surgical procedures to be performed on a non-beating heart with circulatory and respiratory support and is an integral part of cardiac surgery^1^. Despite significant advances, cardiac surgery remains associated with postoperative morbidity and mortality rates ranging from 5% to 75%, depending on patient comorbidities and other factors^2,3^. Intraoperative hypothermia combined with CPB significantly affects patient outcomes^4,5^. While moderate hypothermia is commonly used to increase tolerance to ischemia, recent trends favoring normothermia or mild hypothermia have prompted debate on optimal temperature management. Critical factors include regional temperature monitoring, arterial pH regulation, and acceptable cooling and rewarming gradients^6,7^. The multifaceted nature of hypothermia management in CPB results from its systemic and cellular interactions^8^, necessitating further research into the development of optimal CPB temperature strategies.

CPB has been associated with numerous physiological changes, such as inflammatory cascades, coagulation processes, and organ functions, and also reported to cause significant changes in the plasma metabolomic profile^9^. Metabolomics involves the comprehensive analysis of endogenous and exogenous small molecules and is a powerful tool to assess biochemical changes resulting from genomic, transcriptomic, and proteomic modifications^10–14^. The integration of GC–MS and LC–MS has improved metabolite detection, enhancing the precision of untargeted metabolomic studies^15^. Previous omics studies have investigated CPB from various perspectives. Neonatal and pediatric patients undergoing CPB were studied using proteomic and transcriptomic analyses^16–19^, while urine proteomic studies have provided insights into CPB-associated renal adaptations^20,21^. In addition, CPB-induced inflammatory responses and ischemia-associated metabolic changes were investigated^22–24^. Moreover, recent studies have expanded our understanding of CPB-induced metabolic alterations. Significant changes in purine and carnitine metabolism have been linked to endothelial vascular reactivity, providing new insights into post-CPB physiological responses^25^. Additionally, specific metabolic biomarkers have been proposed for the early prediction and monitoring of acute renal failure following CPB, offering potential clinical utility^26^. It has been applied to assess post-surgical delirium in CPB patients^27^ and to evaluate differences in metabolomic profiles between on-pump and off-pump coronary artery bypass grafting^9^. However, the effects of mild and moderate hypothermia on the specific metabolomic profile in CPB are not yet well understood, representing an important gap in the literature. Addressing this gap is important to improve hypothermia management during CPB.

We performed an untargeted metabolomics analysis by comparing changes in metabolite profiles in mild and moderate hypothermia based on baseline metabolite levels, using blood and urine samples during CPB. This approach allowed us to capture both immediate metabolic fluctuations in plasma and cumulative systemic effects in urine. Our results revealed metabolic alterations and potential biomarker candidates specific to both levels of hypothermia. In our study, we found that moderate hypothermia was generally associated with greater metabolic stress, while mild hypothermia was associated with relatively increased metabolic stability. Our results, when supported by further studies, may contribute to the optimization of CPB and hypothermia protocols for improved clinical outcomes.

## Results

### Patient demographics and baseline clinical characteristics

A total of 32 patients undergoing elective open-heart surgery with cardiopulmonary bypass (CPB) were initially enrolled, with equal allocation to mild hypothermia (32–35 °C, *n* = 16) and moderate hypothermia (26–31 °C, *n* = 16) groups. Each group had an equal number of male and female patients. However, principal component analysis (PCA) identified three outliers due to sample preparation issues, leading to their exclusion (Supplementary File 1.2.1, Figure S1.1). Consequently, the final analyses were conducted on 29 patients (mild hypothermia: 7 males, 8 females; moderate hypothermia: 7 males, 7 females).

PCA was additionally performed as an unsupervised analysis for plasma (T_1_–T_0_ and T_2_–T_0_) and urine (T_2_–T_0_) samples within each hypothermia group to evaluate within-group temporal variation in metabolic profiles (T_0_: before CPB initiation; T_1_: at the minimum hypothermic temperature under cross-clamp conditions; T_2_: one hour after rewarming and CPB termination). The corresponding score plots are presented in Supplementary File S1.2.1 (Figures S1.2 and S1.3).

The mean age and BMI were comparable between groups (*p* = 0.84 and *p* = 0.61 respectively, Table 1). Significant differences were observed in CPB time (145 vs. 193.5 min, *p* = 0.01), cross-clamp time (98 vs. 134.5 min, *p* = 0.01), and lowest rectal temperature (32.5 vs. 29.65 °C, *p* < 0.001). Cardioplegia dose did not differ significantly between groups (*p* = 0.09, Table 1). ICU stay, use of minimally invasive procedures, and autotransfusion rates were similar between the groups (all p-values > 0.05, Table 1). Notably, prior COVID-19 infection was more frequent in the mild hypothermia group (80% vs. 35.71%, *p* = 0.04).

**Table 1:** Patient demographics and baseline clinical characteristics.

Characteristics (Demographics)	Mild hypothermia(*n* = 15)	Moderate hypothermia(*n* = 14)	p values
Ethnicity	White:15(100)	White:14(100)	-
Gender	Male:7,Female:8	Male:7,Female:7	1***
Age (year)	67(63.50,40–77)	66(62.92,45–76)	0.84*
BMI (kg/m²)	26.30(26.75,18.9–32)	27.05(27.13,19.59–33.1)	0.61*
Operative/Clinical Details			
CPB time (minute)	145(145.8,73–213)	193.5(195.92,137–313)	0.01*
Cross clamp time (minute)	98(99.8,48–167)	134.5(141.85,78–240)	0.01*
Lowest rectal temperature (°C)	32.5(32.36,31.3–33.6)	29.65(29.4,27.4–30.5)	< 0.001*
Cardioplegia Dose (L)	1.5(1.56,1.2–2.4)	1.75(1.77,1.3–2.4)	0.09*
ICU (day)	1(1.73,1–4)	1(1.64,1–4)	0.98*
Minimal invasive	2(13.33)	1(7.14)	1**
Cell saver (auto transfusion)	1(6.66)	4(28.57)	0.16**
Smoker (ex or current)	3(20)	6(42.85)	0.24***
Previous COVID-19	12(80)	5(35.71)	0.04***

Data presented as *Mann Whitney U test: median (mean, min-max); **Fisher Exact Test: n(%); ***Pearson Chi-square Test: n(%). BMI, Body mass index; kg/m², kilogram/meter-squared; °C, Degree Celsius; L, Liter; ICU, Intensive care unit.

 Comorbidities (e.g., diabetes mellitus, coronary artery disease, hypertension) and medications (e.g., anticoagulants, beta-blockers, ACE inhibitors) were evenly distributed between groups, with no statistically significant differences (*p* > 0.05 for all comparisons, Supplementary File S1, Table S1.2-S1.3). Similarly, clinical biochemistry parameters measured preoperatively and postoperatively (e.g., AST, creatinine, CRP, albumin) showed no significant intergroup differences (all comparisons had *p* > 0.05, Supplementary File S1, Table S1.1).

## Metabolomics results of plasma samples

Plasma metabolomic analysis identified significant metabolic alterations between mild and moderate hypothermia groups at T_1_-T_0_ and T_2_-T_0_ (Table 2). At T_1_-T_0_, proline and inosine levels showed a greater decrease in the mild hypothermia group compared to the moderate group (*p* = 0.04, *p* = 0.02 respectively). Hexadecenal levels showed a slight increase in both groups (*p* = 0.03), whereas MG(18:1(9Z)/0:0/0:0) levels decreased more in the moderate hypothermia group (*p* = 0.03).

**Table 2:** Significantly changed metabolites between time points T_1_-T_0_ and T_2_-T_0_ in plasma samples.

Time point	Metabolite	Mild hypothermia (*n* = 15)	Moderate hypothermia (*n* = 14)	*p* Values*
T_1_-T_0_	Proline	−0.58 (−0.67, −1.54 – −0.05)	−0.34 (−0.41, −1.01 – −0.05)	0.04
T_1_-T_0_	Inosine	−2.01 (−2.41, −10.38–0.09)	−0.57 (−0.90, −2.39–0.13)	0.02
T_1_-T_0_	Hexadecenal	0.16 (0.15, −0.11–0.34)	0.1 (0.07, −0.19–0.21)	0.03
T_1_-T_0_	MG(18:1(9Z)/0:0/0:0)	−0.22 (−0.72, −4.71–0.26)	−0.57 (−0.54, −1.02 – −0.14)	0.03
T_1_-T_0_	Cer(d18:0/16:0)	−0.002 (0.01, −1.12–0.72)	0.28 (0.5, −0.17–2.06)	0.02
T_2_-T_0_	Cer(d18:0/16:0)	0.12 (0.08, −1.13–0.88)	0.37 (0.45, −0.13–1.26)	0.04
T_2_-T_0_	Cer(t18:0/16:0)	0.16 (0.11, −1.28–0.70)	0.34 (0.37, −0.14–0.92)	0.04

Values indicate metabolite changes between time points (T_1_–T_0_ or T_2_–T_0_), expressed as median (mean, min–max). p-values from *Mann–Whitney U test. MG, monoacylglyceride; Cer, ceramide.

 At T_2_-T_0_, ceramide species (Cer(d18:0/16:0) and Cer(t18:0/16:0)) exhibited a sustained increase in both groups, with a more pronounced elevation in the moderate hypothermia group (*p* = 0.04). These findings indicate distinct metabolic shifts, particularly in amino acid and lipid metabolism, potentially reflecting differential physiological responses to hypothermia levels. A comprehensive list of all detected metabolites, including non-significant findings (*p* > 0.05), can be found in Supplementary File S1, Tables S1.4-S1.5.

**Fig. 1:**
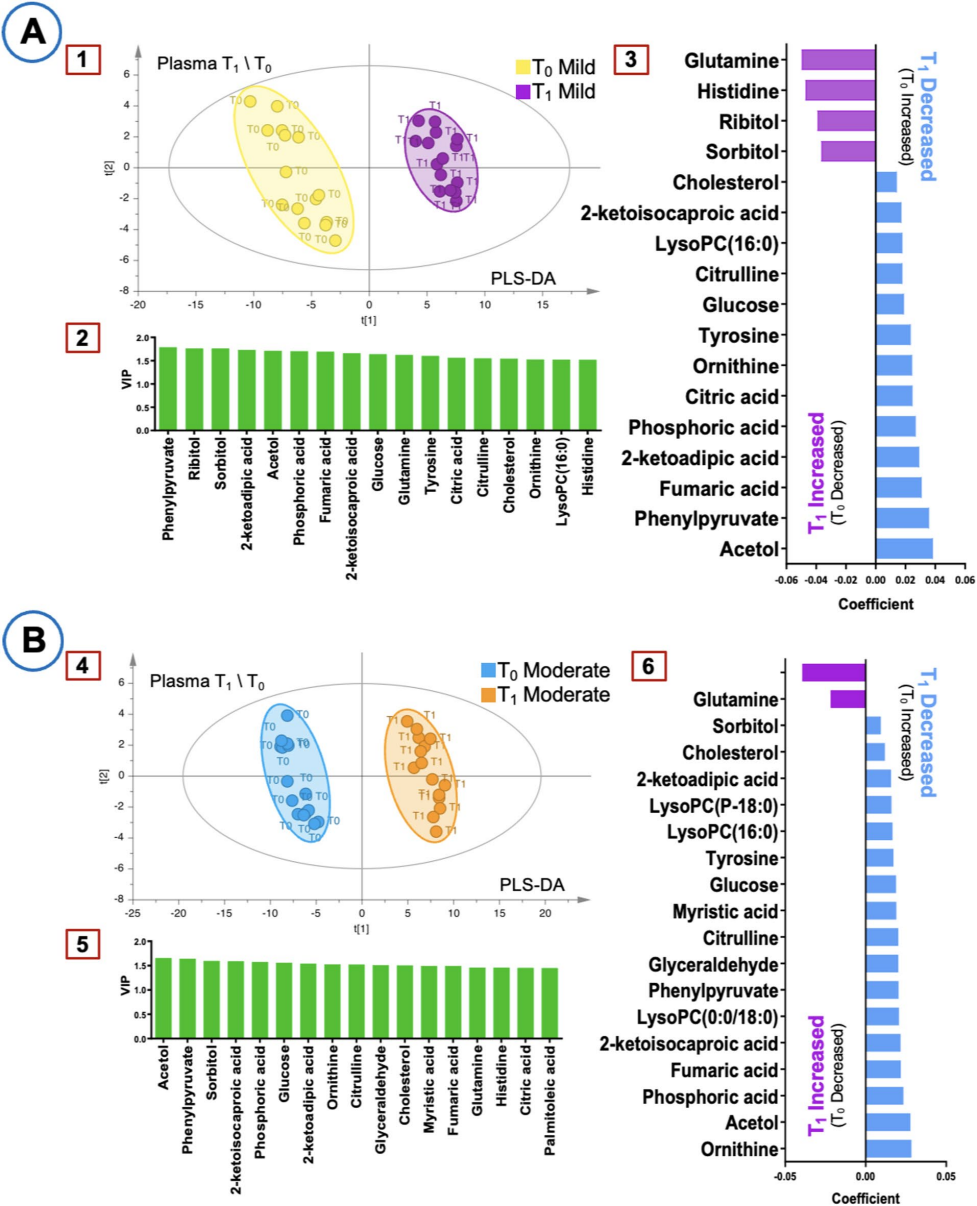
Multivariate analysis of plasma metabolites at T_0_–T_1_. (**A**) Mild hypothermia group (R^2^ = 0.998, Q^2^ = 0.905). (**B**) Moderate hypothermia group (R^2^ = 0.999, Q^2^ = 0.946). Panels 1 and 4: PLS-DA score plots. Panels 2 and 5: VIP plots. Panels 3 and 6: Coefficient plots.

Figure 1 illustrates the Partial Least Squares Discriminant Analysis (PLS-DA) score plots, Variable importance in projection (VIP) plots, and coefficient plots for T_1_-T_0_ comparisons in plasma samples. Sorbitol and ribitol were identified as key metabolites, exhibiting a consistent increase in both groups. In the mild hypothermia group, histidine levels increased, while citric acid levels decreased. In the moderate hypothermia group, significant reductions were observed in LysoPC(P-18:0), myristic acid, glyceraldehyde, and LysoPC(0:0/18:0). These findings highlight distinct metabolic differences between the two hypothermia conditions.

**Fig. 2:**
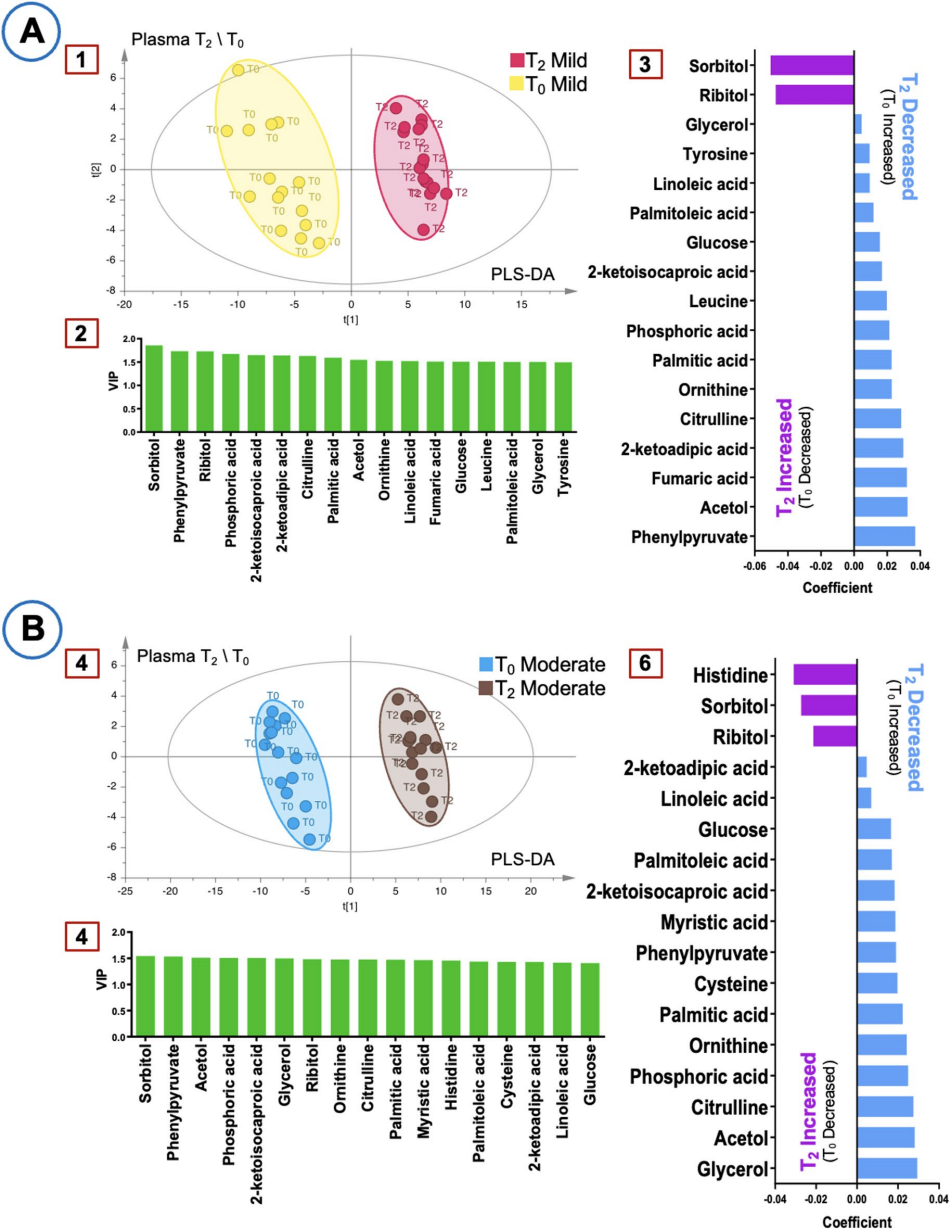
Multivariate analysis of plasma metabolites at T_0_–T_2_. (**A**) Mild hypothermia group (R^2^ = 0.998, Q^2^ = 0.877). (**B**) Moderate hypothermia group (R^2^ = 0.999, Q^2^ = 0.945). Panels 1 and 4: PLS-DA score plots. Panels 2 and 5: VIP plots. Panels 3 and 6: Coefficient plots.

Figure 2 illustrates the PLS-DA score plots, VIP plots, and coefficient plots for T_2_-T_0_ comparisons in plasma samples. Sorbitol and ribitol remained key metabolites, showing a consistent increase in both groups. Unlike T_1_-T_0_, histidine levels increased in the moderate hypothermia group. Tyrosine, leucine, and fumaric acid levels decreased in the mild hypothermia group, whereas myristic acid was the only significantly decreased metabolite in the moderate hypothermia group. These findings indicate distinct metabolic profiles emerging at T_2_-T_0_, further differentiating the two hypothermia groups.

Collectively, these findings demonstrate time-dependent metabolic alterations in plasma, with distinct patterns observed between mild and moderate hypothermia groups. While sorbitol and ribitol consistently increased in both groups, other metabolic shifts —including changes in histidine, fatty acids, and phospholipid derivatives— varied by hypothermia level and time point. These results provide a comprehensive overview of plasma metabolomic responses to hypothermia.

## Metabolomics results of urine samples

Urinary metabolomic analysis at T_2_-T_0_ identified significant metabolic differences between mild and moderate hypothermia groups (Table 3). C17-sphinganine levels increased in both groups, with a more pronounced rise in the moderate hypothermia group (*p* = 0.02). Similarly, 2-hydroxybutyric acid levels decreased in both groups, with a greater reduction in the mild hypothermia group (*p* = 0.01).

**Table 3:** Significantly changed metabolites between time points in urine samples (T_2_-T_0_).

Metabolite	Mild hypothermia (*n* = 15)	Moderate hypothermia (*n* = 14)	*p* Values*
C17-sphinganine	0.65 (0.58, −0.45–1.89)	0.93 (0.88, 0.38–1.39)	0.02
2-Hydroxybutyric acid	−1.39 (−2, −5.46 – −0.08)	−0.47 (−0.83, −4.11–0.19)	0.01
3-methyl-2-Oxobutanoic acid	−1.35 (−1.98, −5.30 – −0.18)	−0.4 (−0.9, −3.97–0.28)	0.02
Acetyl-serine	−1.58 (−1.81, −4.20 – −0.35)	−0.93 (−1.17, −2.68 – −0.33)	0.04
Oxalacetic acid	−1.2 (−1.58, −4.93–0.38)	−0.5 (−0.67, −2.38–0.13)	0.02

Values indicate metabolite changes between time points (T_2_–T_0_), expressed as median (mean, min–max). p-values from *Mann-Whitney U test.

 Additional significant changes were observed in 3-methyl-2-oxobutanoic acid, acetyl-serine, and oxaloacetic acid levels, with distinct patterns between groups. 3-methyl-2-oxobutanoic acid and acetyl-serine exhibited greater reductions in the mild hypothermia group (*p* = 0.02, *p* = 0.04, respectively), whereas oxaloacetic acid showed a more substantial decrease in the mild hypothermia group compared to the moderate group (*p* = 0.02). Please see Supplementary File S1, Table S1.6, for a comprehensive list of all detected metabolites, including non-significant findings (*p* > 0.05).

**Fig. 3:**
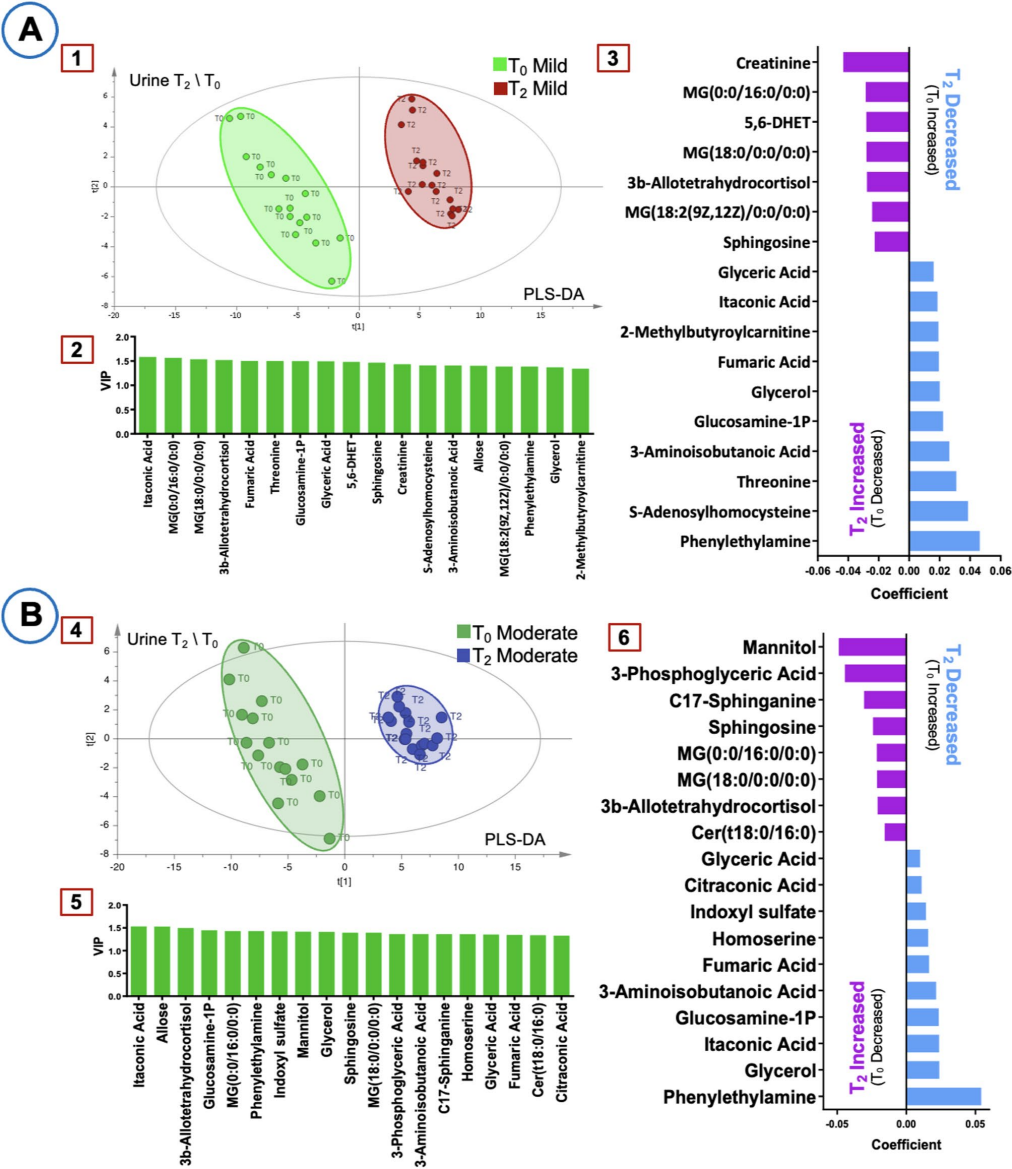
Multivariate analysis of urine metabolites at T_0_–T_2_. (**A**) Mild hypothermia group (R^2^ = 0.987, Q^2^ = 0.939). (**B**) Moderate hypothermia group (R^2^ = 0.984, Q^2^ = 0.939). Panels 1 and 4: PLS-DA score plots. Panels 2 and 5: VIP plots. Panels 3 and 6: Coefficient plots.

Figure 3 illustrates the PLS-DA score plots, VIP plots, and coefficient plots for T_2_-T_0_ comparisons in urine samples. In the mild hypothermia group, creatinine, 5,6-Dihydroxy-8Z,11Z,14Z-eicosatrienoic acid (5,6-DHET), and MG(18:2(9Z,12Z)/0:0/0:0) levels increased, whereas threonine and S-adenosylhomocysteine levels decreased. In the moderate hypothermia group, mannitol, 3-phosphoglyceric acid, C17-sphinganine, and Cer(t18:0/16:0) exhibited significant increases, while indoxyl sulfate and homoserine levels decreased. These findings highlight distinct urinary metabolic shifts at T_2_-T_0_, differentiating the two hypothermia conditions.

Metabolite comparisons for T_1_-T_0_ and T_2_-T_0_ in plasma and urine samples of mild and moderate hypothermia groups were visualized using volcano plots. Statistically significant metabolites were identified based on a fold change (FC) > 2 and an FDR-adjusted p-value < 0.05. The results are presented in Supplementary File S1.2.2, Figure S1.4, and related findings are provided in Supplementary_File_S3_Volcano_Plots_and_Pathway_Analysis.xlsx. In addition, pathway analyses were performed using the significant metabolites identified from the volcano plot analysis (FC > 2, FDR-adjusted *p* < 0.05); the results are summarized in Supplementary File S1.2.3 and Supplementary_File_S3_Volcano_Plots_and_Pathway_Analysis.xlsx, Figure S1.5.

## Discussion

In this study, we investigated comprehensively the effects of mild and moderate hypothermia on plasma and urine metabolomic profiles during CPB using untargeted analyses with GC-MS and LC‐qTOF‐MS‐based instruments. The integration of GC-MS and LC-MS has improved metabolite detection, enhancing the precision of untargeted metabolomic studies^15^. Although several metabolomic studies have explored CPB‐related changes, the specific impacts on the plasma and urine metabolome of mild versus moderate hypothermia have not been fully elucidated. Our analysis revealed comparable baseline demographic and clinical characteristics between the groups (Table 1). While demographic and clinical differences such as CPB duration and COVID‐19 history were observed, these factors did not significantly influence pre‐ and post‐operative biochemistry, allowing a clearer focus on metabolic differences driven by hypothermia. However, the differences in prior COVID-19 history between the groups is acknowledged as a study limitation. Although this variable was not included in multivariate models due to the small cohort size and risk of overfitting, residual metabolic effects in some individuals cannot be entirely ruled out. Although CPB and cross-clamp durations were not included in the statistical models, these temporal differences may have contributed to the observed metabolic variations and are considered among the study limitations.

The primary goal of hypothermia during CPB is to protect organs by reducing metabolic rate and oxygen consumption^28,29^. Hypothermia was generally associated with reduced plasma metabolic activity; however, sorbitol and ribitol levels increased at both T_1_ and T_2_, likely reflecting polyol pathway activation and distinct metabolic adaptations (Figs. 1 and 2). While plasma metabolomics reflects immediate metabolic shifts in response to hypothermia and CPB, urine provides insights into cumulative metabolic processing over time, making these biofluids complementary in understanding overall metabolic adaptations.

Building on these observations, urine metabolites offer valuable insights as they represent end products of both normal and pathological cellular processes^30,31^. With lower complexity and protein content than other biofluids, urine requires minimal pre-treatment, making it particularly suitable for metabolomic studies^30,32^. These characteristics contribute to stable and consistent metabolite detection, resulting in more reliable metabolic profiles. Supporting this, PLS‐DA analysis revealed a clearer separation between mild and moderate hypothermia groups in urine samples (R^2^ and Q^2^ values > 0.9), emphasizing the reliability of urine metabolomics in detecting hypothermia‐induced changes (Fig. 3).Furthermore, this study distinguishes the immediate effects observed in plasma from the cumulative changes captured in urine. Such an approach not only enhances our understanding of hypothermia-induced metabolic adaptations but also provides novel insights into optimizing perioperative hypothermia management.

Analysis of plasma samples revealed that, according to Mann-Whitney analyses (Table 3), proline and inosine levels were significantly decreased at T_1_ compared to baseline (T_0_) in the mild hypothermia group, whereas Cer(d18:0/16:0) levels were significantly elevated and MG(18:1(9Z)/0:0/0:0) levels were significantly decreased in the moderate hypothermia group. Inosine, a key nucleoside in purine biosynthesis, gene translation, and RNA modulation, plays roles in neuroprotection and immunomodulation^33,34^. The observed lower inosine levels in the mild hypothermia group compared to the moderate group may indicate altered purine metabolism; further research is required to determine potential implications for neuroprotection. Similarly, the significant decrease in proline levels in the mild hypothermia group at T_1_ may reflect alterations in protein or amino acid metabolism^35^; however, further targeted studies are needed to confirm broader metabolic implications. Elevated hexadecenal levels in the mild hypothermia group may indicate changes in lipid-related pathways, potentially including sphingolipid-related processes (e.g., signaling or apoptosis)^36^, though further investigation is required. This increase may reflect a metabolic adaptation to hypothermic stress, warranting further investigation. In contrast, MG(18:1(9Z)/0:0/0:0) levels decreased more significantly in the moderate hypothermia group, suggesting an impact on lipid metabolism, particularly in the synthesis of triacylglycerols^37^; this pronounced decrease may indicate altered thermogenesis. Additionally, Cer(d18:0/16:0) levels increased significantly in the moderate hypothermia group compared to T_0_, indicating enhanced lipid metabolic activity and potential involvement in oxidative stress and apoptosis^38^. The increase was more prominent in the moderate hypothermia group, highlighting potential differences in stress responses between the groups. At T_2_, following rewarming and CPB termination, notable changes were observed only in the moderate hypothermia group, with significant increases in Cer(d18:0/16:0) and Cer(t18:0/16:0) levels, reflecting increased lipid metabolic activity and cellular stress responses (Table 3).

Further plasma analysis demonstrated that pairwise comparisons between mild and moderate hypothermia groups yielded R^2^ and Q^2^ values > 0.9, indicating highly reliable models. Intraoperative hyperglycemia, common in cardiac surgery, leads to glucose converting into polyols such as sorbitol and ribitol via the polyol pathway^39–41^. The observed increase in sorbitol at T_1_ and T_2_ may partially result from its exogenous administration via a sorbitol-containing enema pre-surgery, rather than being solely driven by metabolic changes. Additionally, sorbitol is known to be absorbed from the intestine^42^, which may have contributed to its elevated plasma levels. In contrast, elevated ribitol levels likely reflect polyol pathway activation due to surgical stress (Figs. 1 and 2). At T_1_, plasma glutamine increased in both groups, indicating higher metabolic demand due to surgical stress^43^. Histidine levels showed distinct temporal variations between groups, likely influenced by the histidine-containing Bretschneider solution used for myocardial protection^44,45^. Fumaric acid levels decreased at T_1_ in the moderate hypothermia group and at T_2_ in the mild group, suggesting differential effects of hypothermia on cellular energy balance^46^. Myristic acid also decreased, implying changes in lipid metabolism^47^, and citric acid decreased in the mild group at T_1_, indicating impacts on the Krebs cycle and energy production^48,49^. Moreover, moderate hypothermia at T_1_ resulted in significant decreases in glyceraldehyde, LysoPC(P-18:0), and LysoPC(0:0/18:0), suggesting effects on carbohydrate metabolism and oxidative stress response^50,51^. These findings collectively underscore the impact of moderate hypothermia on lipid metabolism, energy production, and cellular stress responses, providing insights for optimizing perioperative management (Figs. 1 and 2).

**Fig. 4:**
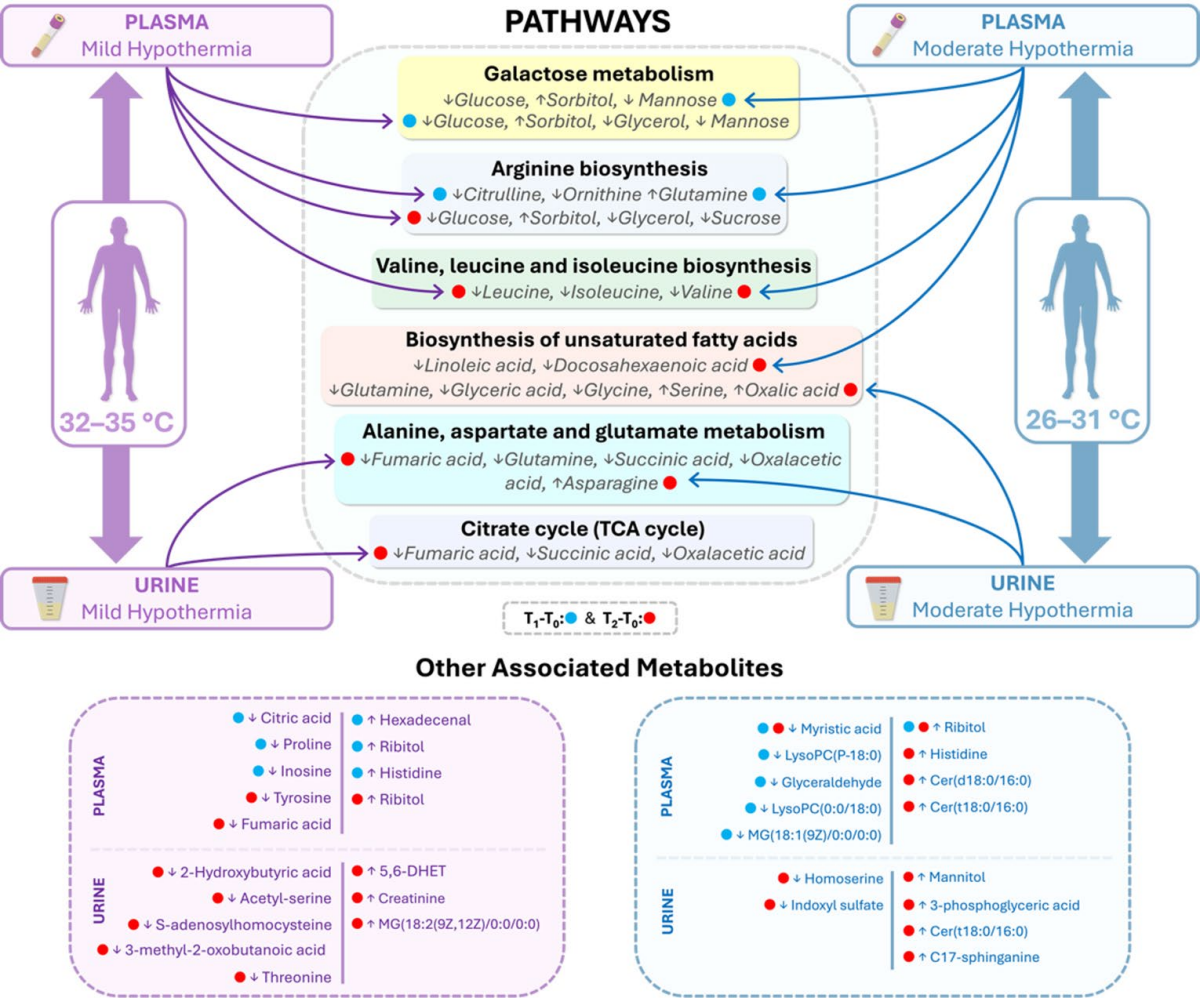
Schematic overview of significantly altered pathways and their associated identified metabolites under mild (32–35 °C) and moderate (26–31 °C) hypothermia; each metabolite is presented with arrows (↑/↓) indicating the direction of relative-abundance changes. Blue and red dots indicate time points (T_1_-T_0_ and T_2_-T_0_, respectively); curved connectors (purple = mild, blue = moderate) represent links between groups and affected pathways.

Additionally, the results of our pathway analyses revealed important biological signals that support our findings. In plasma samples, both the mild and moderate hypothermia groups exhibited significant alterations in galactose metabolism and arginine biosynthesis at the T_1_–T_0_ time point. At T_2_–T_0_, valine, leucine, and isoleucine biosynthesis and galactose metabolism were prominently affected in the mild hypothermia group, while valine, leucine, and isoleucine biosynthesis and biosynthesis of unsaturated fatty acids were distinctly altered in the moderate hypothermia group. Similarly, previous CPB studies have reported significant changes in arginine and amino acid metabolism following cardiopulmonary bypass^9,26^. In urine samples, especially at the T_2_–T_0_ time point, alanine, aspartate, and glutamate metabolism and the citrate cycle were notably affected in the mild hypothermia group, whereas alanine, aspartate, and glutamate metabolism and glyoxylate and dicarboxylate metabolism were significantly altered in the moderate hypothermia group. Metabolomic analyses focusing on urine have also demonstrated marked changes in amino acid and energy metabolism pathways following CPB^21^. Overall, our findings indicate that the observed metabolite alterations are predominantly concentrated in pathways related to amino acid and lipid metabolism, and that different degrees of hypothermia induce distinct biochemical adaptations. Thus, our results highlight the fundamental differences between mild and moderate hypothermia, particularly in terms of energy metabolism and cellular stress responses (Supplementary File S1.2.3, Figure S1.5, and Supplementary File S3). The overall metabolite and pathway alterations, categorized by group and time point, are summarized in Fig. 4.

Mann-Whitney analyses of urine samples revealed that C17-sphinganine levels were significantly increased compared to T_0_ in the moderate hypothermia group, indicating a stronger influence on cell membrane remodeling and stress responses. C17‐sphinganine, as an essential sphingolipid, plays a key role in membrane integrity; and its elevation under moderate hypothermia suggests a shift in sphingolipid metabolism, possibly linked to inflammatory processes^52,53^ (Table 3). This finding, along with the overall multivariate analysis results, further confirms the role of C17‐sphinganine in hypothermia-induced metabolic adaptations (Fig. 3B). In contrast, mild hypothermia resulted in significant decreases in several urine metabolites (Table 3). One of the most notable reductions was observed in 2-hydroxybutyric acid a by-product of amino acid catabolism and glutathione synthesis that serves as an early biomarker of lipid oxidation and oxidative stress. Reduced 2-hydroxybutyric acid levels in the mild hypothermia group may indicate modifications in metabolic stress pathways or potentially beneficial alterations in glucose homeostasis^54^; however, these interpretations warrant further validation through targeted investigations. Another key metabolite that decreased under mild hypothermia was N-acetylserine, which has been implicated in renal function and is considered a potential biomarker for tubular dysfunction^55^. The decline in N-acetylserine under mild hypothermia may reflect differences in renal-associated metabolic responses between hypothermia levels (potentially including protective effects); however, further research is warranted to evaluate clinical significance. Similarly, a reduction in 3-methyl-2-oxobutanoic acid metabolite reported to reduce uremic toxicity and alleviate symptoms in chronic kidney disease was observed in the mild hypothermia group^56,57^. This implies that the metabolic response to mild hypothermia may align with previously described protective effects against metabolic stress. Furthermore, the decrease in oxaloacetate in the mild hypothermia group might reflect alterations in central energy metabolism (including the Krebs cycle and gluconeogenesis)^12,58^; however, these findings should be validated through targeted metabolomic analyses. These metabolic shifts suggest that mild hypothermia may promote a more stable energy metabolism compared to the stronger metabolic stress observed under moderate hypothermia.

Multivariate analysis (PLS-DA, VIP, and coefficient analysis) demonstrated highly reliable models for distinguishing between the mild and moderate hypothermia groups, with R^2^ and Q^2^ values exceeding 0.9 (Fig. 3). In the mild hypothermia group, the observed moderate increase in creatinine levels suggests potentially lower renal stress compared to moderate hypothermia, as creatinine is a key marker of renal function^37^. Additionally, the increase in 5,6-DHET under mild hypothermia indicates a reduced impact on renal vascular function, supporting improved vascular regulation; 5,6-DHET, derived from arachidonic acid oxidation, plays a role in renal epithelial transport and vascular homeostasis^59,60^ (Fig. 3A). At T_0_, lower acylcarnitine levels in the mild hypothermia group further suggest better preservation of lipid metabolism particularly in fatty acid oxidation and energy production (beta-oxidation)^61^ (Fig. 3A). The decrease in threonine levels under mild hypothermia may reflect differences in amino acid or lipid metabolism compared to moderate hypothermia, potentially involving effects on thermogenic gene expression^62,63^. This finding is depicted in Fig. 3A; however, further studies are warranted to validate these observations. Moreover, when compared to baseline (T_0_), mild hypothermia was associated with lower S-adenosylhomocysteine levels, suggesting a reduced systemic inflammatory response and a lower risk of myocardial ischemia relative to moderate hypothermia. Elevated S-adenosylhomocysteine has been linked to SIRS, sepsis, and increased mortality in critically ill patients^64–66^, and it is associated with hypoxia-driven adenosine productiona potential marker of myocardial ischemia^64,67^ (Fig. 4B).

In the moderate hypothermia group, notable increases were observed in 3-phosphoglyceric acid and Cer(t18:0/16:0) levels compared to T_0_ (Fig. 3B). The marked elevation in mannitol was expected, as it was administered as an osmotic diuretic during CPB and its levels correlate with longer cross-clamp and CPB durations (Fig. 3B). Furthermore, moderate hypothermia led to an increase in 3-phosphoglyceric acid, a key intermediate in glycolysis, highlighting potential metabolic shifts in energy production pathways^37^ (Fig. 3B). The pronounced increase in ceramide levels observed under moderate hypothermia suggests alterations in lipid metabolism possibly related to cellular stress^38^; however, further mechanistic studies are necessary to confirm associations with apoptosis and oxidative stress (Fig. 3B). Moreover, moderate hypothermia resulted in greater reductions in indoxyl sulfate, homoserine, and citraconic acid levels compared to mild hypothermia. Indoxyl sulfate, a uremic toxin associated with oxidative stress and cardiovascular risk in chronic kidney disease^68^, decreased under moderate hypothermia, potentially reflecting a protective metabolic shift against oxidative damage (Fig. 3B). Similarly, the significant decline in homoserine levels suggests alterations in protein synthesis and amino acid metabolism, as homoserine an intermediate in methionine and threonine metabolism may indicate temperature-driven metabolic adjustments^37,69^. (Fig. 3B). Finally, the notable decrease in citraconic acid, an inhibitor of fumarate reduction, highlights potential impacts on cellular energy production and mitochondrial function^70,71^ (Fig. 3B).

These findings provide novel insights into the metabolic signatures of mild and moderate hypothermia and, when supported by further research, may contribute to optimizing CPB protocols. Additional targeted metabolomic studies could aid in identifying potential biomarkers for refined temperature management strategies during cardiac surgery.

In conclusion, this study provides a comprehensive analysis of the differential metabolic effects of mild and moderate hypothermia during CPB. Mild hypothermia was associated with a favorable metabolic profile, marked by reduced cellular stress and improved energy metabolism, as reflected in plasma and urine analyses. Conversely, moderate hypothermia, necessary in more complex procedures, presented heightened metabolic stress, emphasizing the need for careful management. In addition, COVID-19 history in mild hypothermia patients may contribute to specific metabolic patterns observed, although this requires further studies. These findings underscore the importance of tailoring hypothermia strategies to optimize metabolic outcomes in CPB.

Although our untargeted approach does not permit a direct linkage between individual metabolite changes and clinical outcomes, the consistent differences observed in metabolites related to energy production, oxidative stress and renal-associated metabolomic responses suggest distinct metabolic trajectories under mild and moderate hypothermia. These preliminary findings lay the groundwork for targeted validation studies that couple metabolite signatures with organ-specific endpoints and postoperative outcomes. Such efforts may ultimately support biomarker-guided refinement of hypothermia strategies, with the goal of minimizing metabolic stress and preserving organ function during cardiopulmonary bypass.

Future studies should seek to validate these findings in larger and more diverse cohorts, with a focus on long-term metabolic implications and clinical outcomes. Investigating the long-term outcomes of these metabolic changes and their interplay with cardioplegia solutions and comorbidities like COVID-19 will further refine CPB protocols. Identifying and validating key metabolites as biomarkers offers promising avenues for developing personalized hypothermia strategies, ultimately improving patient outcomes.

While these findings provide valuable insights, certain limitations must be acknowledged. The relatively limited number of samples and the single-site design may constrain the applicability of the results. The absence of long-term follow-up limits our understanding of the sustained metabolic effects and their clinical significance. Future studies exploring the role of different cardioplegia solutions and COVID-19 interactions will provide a more comprehensive understanding. Addressing these limitations will enhance the reliability and clinical utility of hypothermia protocols in cardiac surgery.

## Materials and methods

### Study cohort and design

This prospective, randomized study included 32 patients undergoing elective open-heart surgery with CPB, assigned to mild (32–35 °C, *n* = 16) or moderate (26–31 °C, *n* = 16) hypothermia groups. The rewarming temperature was maintained below 37.5 °C. A priori statistical power analysis was conducted using G*Power software (version 3.1.9.7). Based on an expected effect size of 1.05, a significance level (α) of 0.05, and a power of 0.80, the required total sample size was determined to be 32 participants (16 per group). Therefore, 32 patients were initially recruited. Three patients were excluded based on a predefined PCA-based outlier criterion, as their metabolomic profiles consistently fell outside the 95% confidence interval in both plasma and urine datasets (see Supplementary File S1, Figure S1.1). Eligible patients (aged 40–80 years, BMI < 35, stable organ function) underwent elective surgery with HTK cardioplegia. Exclusion criteria included prior open-heart surgery, ongoing infections, and respiratory or extracorporeal life support (detailed in Supplementary File S2.1). All patients underwent anesthesia with the same standardized institutional protocol, including consistent agents and an average interval of 45–55 min between anesthesia induction and T_0_ sampling, as detailed in Supplementary File S2.6.

This study was conducted in accordance with the Declaration of Helsinki and was approved by the Ankara University Faculty of Medicine Human Research Ethics Committee (Date: 13 January 2022, No: I01-12–22). Only individuals who provided written and oral informed consent were included in the study. Patients scheduled for elective surgery at the Department of Cardiovascular Surgery at Ankara University Faculty of Medicine were included. To minimize variability, all patients were managed using the same oxygenator, prime solution type and volume, and tubing set configuration. Details regarding the CPB circuit and perfusion parameters are provided in Supplementary File S2.5.

**Fig. 5:**
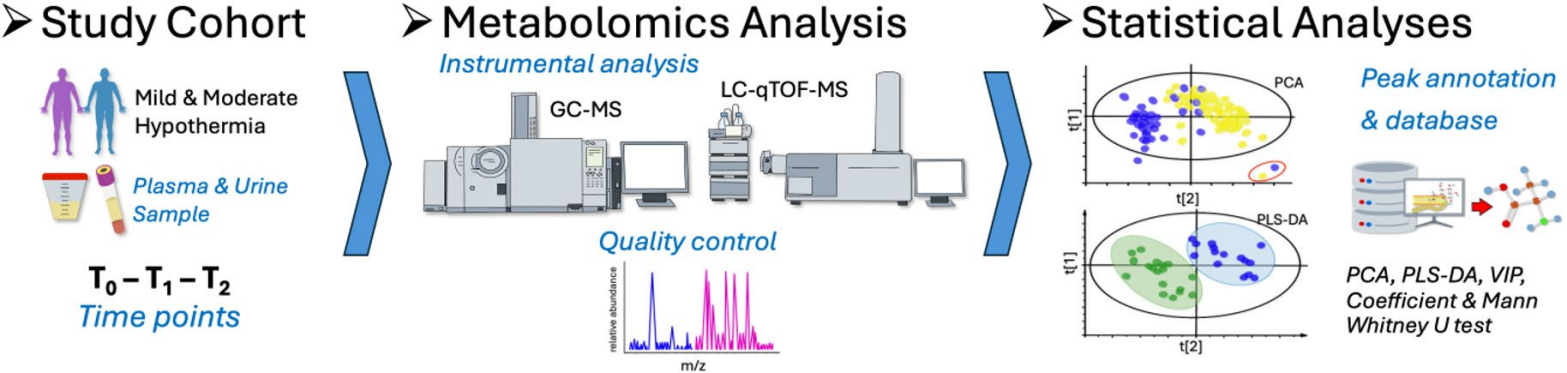
Study workflow overview.

Blood and urine samples were collected at predefined time points: before CPB initiation (T_0_), at the minimum hypothermic temperature under cross-clamp conditions (T_1_), and one hour after rewarming and CPB termination (T_2_). Plasma samples were obtained at T_0_, T_1_, and T_2_, while urine samples were collected at T_0_ and T_2_ (detailed in Supplementary File S2.2). To standardize hydration status across patients, a uniform preoperative fasting protocol—including fluid restriction—was applied for at least 8 h prior to surgery. The overall workflow of the study is illustrated in Fig. 5.

## Metabolomics analysis and sample Preparation

Metabolomic analyses were performed using LC-qTOF-MS (Agilent 6530, California, USA) and GC-MS (Shimadzu QP2010 Ultra, Kyoto, Japan). Plasma and urine samples were processed following standardized protocols, including methanol-based protein precipitation, urease incubation (urine), and derivatization (GC-MS). QC samples were prepared by pooling aliquots from all samples. Details on sample preparation, derivatization, and chromatographic conditions are provided in Supplementary File S2.3.

## Data processing and statistical analysis

Metabolomic data were processed using MS-DIAL (version 4.92) for peak detection, deconvolution, and retention time alignment. Normalization was performed based on the total peak area of the total ion chromatogram. Metabolite identification for GC-MS was carried out using retention index-based Fiehn and Golm libraries, while LC-qTOF-MS metabolite identification was conducted by comparing MS/MS spectra from QC samples (collected at collision energies of 10, 20, and 40 eV) with open-access spectral libraries (positive and negative mode, RIKEN). Following data processing and curation, a total of 137 metabolites were identified in plasma at T_1_-T_0_, 119 metabolites at T_2_-T_0_, and 101 metabolites in urine at T_2_-T_0_. These identified metabolites were further evaluated for statistically significant alterations (see Supplementary file S1, Tables S1.4-S1.6).

Given the data complexity, we have conducted multivariate statistical analyses. We have combined data matrices from urine and plasma samples after data curation and transferred these to SIMCA 14.1 for PCA and PLS-DA. PCA plots were used to detect systematic errors and outliers, while PLS-DA plots visualized group differentiation in both global and pairwise comparisons. VIP plots and coefficient plots from PLS-DA served to identify significantly altered metabolites and their direction of change. We have used R^2^and Q^2^ values to assess model reliability.

We have utilized R software (R Core Team, 2024) for statistical analyses. The Wilcoxon test was applied to compare paired time points (T_1_-T_0_, T_2_-T_0_) for detecting significantly altered metabolites. We have applied the Mann-Whitney U test to compare two independent groups (mild and moderate) in terms of the change between post and pre-operative biochemical parameters and time-independent continuous variables (age, bmi, etc.). Descriptive statistics are reported as median (mean, minimum - maximum). For categorical data analysis, Pearson’s chi-square test and Fisher’s exact test were applied based on contingency table values. We have used Fisher’s exact test where any expected cell count was below 5; otherwise opting for Pearson’s chi-square test,. A significance threshold of *p* < 0.05 was applied for all statistical evaluations. Supplementary File S2.4 provides further details on data preprocessing, metabolite identification, and statistical validation.

## Supplementary Information

Below is the link to the electronic supplementary material.

Supplementary Material 1

Supplementary Material 2

Supplementary Material 3

## Data Availability

The datasets generated and analyzed during the current study are available in the Supplementary Information files. In addition, the raw metabolomics data have been deposited in the MassIVE repository and are publicly available under the accession number MSV000098691. The dataset can be accessed at https://doi.org/10.25345/C5QR4P38M.
