# Supplementary material for: Differential metabolomic signatures in plasma and urine under mild and moderate hypothermia during cardiopulmonary bypass
Source: Sci Rep. 2025 Nov 20;15:41079. doi: 10.1038/s41598-025-24913-9 (PMC12634694; doi:10.1038/s41598-025-24913-9)
Supplement: Supplementary file 1 — Supplementary Material 1 [file 41598_2025_24913_MOESM1_ESM.pdf]

S1.1 Additional Clinical and Demographic Data

S1.1.1 Clinical Biochemistry Parameters

Preoperative and postoperative clinical biochemistry parameters were measured in both mild and moderate hypothermia groups. The table presents median values, interquartile ranges, and p-values for key biochemical markers, including AST, creatinine, CRP, and albumin. No statistically significant differences were observed between the groups ($p > 0.05$).

Table S1.1 Changes in Clinical Biochemistry Parameters Before and After Surgery in Mild and Moderate Hypothermia Groups

Clinical biochemistry parameters	Mild Hypothermia (n=15) median (mean, min-max)	Moderate Hypothermia (n=14) median (mean, min-max)	p Values
AST (U/L)	80 (79.8, -8 – 196)	119 (181.85, 16 – 822)	0.12
Creatinine (mg/dL)	0,13 (0.09, -0,27 – 0.54)	0.17 (0.29, -0.18 – 1.10)	0.34
GFR (mL/min/1,73m2)	-13 (-6.4, -26 – 20)	-13 (-14.28, -50 – 16)	0.26
CRP (mg/L)	48.2 (45, -3.4 – 71.4)	36.85 (34.76, 13.3 – 65)	0.09
ALT (U/L)	8 (9.4, -30 – 38)	8.5 (87.07, -10 – 709)	0.41
LDH (U/L)	255 (287.4, -33 – 608)	346 (441.85, -8 – 1009)	0.07
Albumin (g/dL)	-10.5 (-8.98, -15.3 – -2.3)	-11.85 (-11.84, -18.5 – 4.6)	0.11
INR (sec)	0.16 (0.12, -0.29 – 0.35)	0.28 (0.2, -0.61 – 0.76)	0.19
BUN (mg/dL)	5 (3.93, -6 – 13)	2 (4.35, -3 – 26)	0.46

AST, Aspartate aminotransferase; GFR, Glomerular filtration rate; CRP, C-reactive protein; ALT, Alanine aminotransferase; LDH, Lactate dehydrogenase; INR, International normalized ratio; Bun, Blood urea nitrogen; U/L, Units per liter; mg/dL, Milligrams/deciliter; mL/min/1,73m2, 1 (milliliter / minute) / (1,73 (square meters)); g/dL, gram/deciliter; sec, second.

S1.1.2 Comorbidities

The presence of comorbidities in patients undergoing mild and moderate hypothermia was recorded. The distribution of comorbidities, including diabetes, coronary artery disease, hypercholesterolemia, and hypertension, among the groups is presented in **Table S1.2** No statistically significant differences were observed between the groups ($p > 0.05$).

Table S1.2 Comorbidities of Patients Undergoing Mild and Moderate Hypothermia During Cardiopulmonary Bypass

Comorbidities	Mild Hypothermia (n=15) n(%)	Moderate Hypothermia (n=14) n(%)	p Values
Diabetes mellitus	5 (33.33)	1 (7.14)	0.16*
Coronary artery disease	2 (13.33)	5 (35.71)	0.21*
Hypercholesterolemia	3 (20)	2 (14.28)	1*
Atrial fibrillation	4 (26.66)	3 (21.42)	1*
Hyperthyroidism	1 (6.66)	0 (0.00)	1*
Hypothyroidism	3 (20)	2 (14.28)	1*
Rheumatisms	2 (13.33)	1 (7.14)	1*
COPD	1 (6.66)	1 (7.14)	1*
Hypertension	10 (66.66)	7 (50)	0.59**

Data presented as * Fisher Exact Test: n(%); **Pearson Chi-square Test: n(%).COPD, Chronic obstructive pulmonary disease;

S1.1.3 Medications

Medications used by patients prior to surgery were documented. The frequency and distribution of anticoagulants, antiplatelets, ACE inhibitors, and other commonly prescribed drugs are summarized in **Table S1.3** No statistically significant differences were found between the groups ($p > 0.05$).

Table S1.3 Comorbidities of Patients Undergoing Mild and Moderate Hypothermia During Cardiopulmonary Bypass

Medications	Mild Hypothermia (n=15) n(%)	Moderate Hypothermia (n=14) n(%)	p Values
Anticoagulant	3 (33.33)	4 (28.57)	1**
Antiplatelet	6 (40)	5 (35.71)	1**
ACE Inhibitor	5 (33.33)	5 (35.71)	1**
Betablocker	7 (46.66)	11 (78.57)	0.16**
Diuretic	5 (33.33)	6 (42.85)	0.88**
Ca^{+2} channel blocker	3 (20)	1 (7.14)	1*
AT2 Inhibitor	5 (33.33)	2 (14.28)	0.16*
Metformin	3 (20)	1 (7.14)	0.59*
Dpp4	2 (13.33)	0 (0)	0.48*
Insulin	1 (6.66)	0 (0)	1*
Atorvastazin	1 (6.66)	4 (28.57)	0.16*
Antirheumatic	1 (6.66)	1 (7.14)	1*
Antihyperthyroid	1 (6.66)	0 (0.00)	1*
Antihypothyroid	3 (20)	2 (14.28)	1*
Data presented as * Fisher Exact Test: n(%); **Pearson Chi-square Test: n(%). AT2, Angiotensin II receptor; ACE, Angiotensin-converting enzyme; DPP4, Dipeptidyl peptidase-4.			

S1.2 Data analysis of GC-MS and LC-qTOF-MS based metabolomics analyzes

S1.2.1 Principal Component Analysis (PCA)

Principal Component Analysis (PCA) was conducted to evaluate the clustering and variability within the metabolomic datasets for both plasma and urine samples. This analysis was performed to identify potential outliers that could affect the robustness of the study results. **Figure S1.1A** illustrates the PCA score plot for plasma samples across the three time points (T_0, T_1, T_2). The plot demonstrates clear clustering patterns for each time point, indicating distinct metabolomic profiles at different stages of hypothermia. However, the outlier samples from three patients, highlighted with red circles, exhibited values that deviated significantly from their respective clusters. **Figure S1.1B** represents the PCA score plot for urine samples at

T_0 and T_2. Similar clustering patterns were observed, with well-defined separation between the time points. The same outlier samples, highlighted with a red circle, were consistently observed across both plasma and urine datasets. Based on these findings, the three outlier samples were excluded from further analyses to maintain the integrity and reliability of the results. Details of the excluded samples and their respective positions on the PCA plots are provided in **Figure S1.1**.

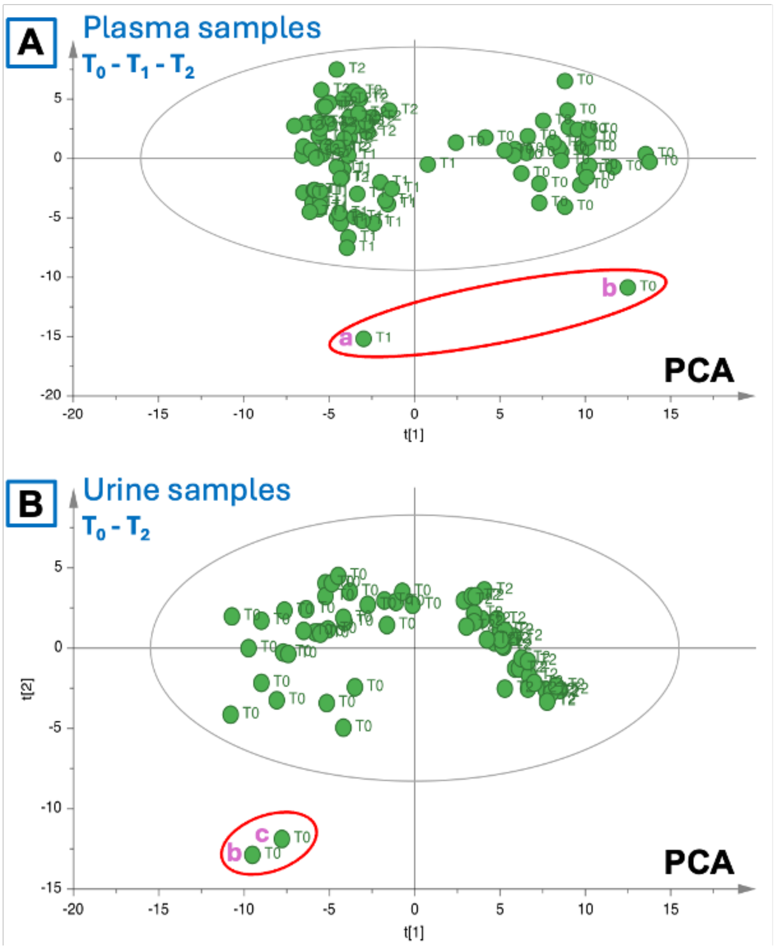

Figure S1.1 PCA score plot. A) Plasma samples (T_0, T_1, T_2). B) Urine samples (T_0, T_2). Outlier samples are highlighted with a red circle. The points labeled as **a**, **b**, and **c** represent three patients associated with the outlier samples.

Supervised methods (e.g., PLS-DA) in metabolomics may be prone to overfitting due to the use of predefined group categorizations. Therefore, PCA was performed as an unsupervised analysis. In plasma samples, unsupervised PCA was applied to compare T_1-T_0 and T_2-T_0 within each hypothermia group (**Figure S1.2**). Similarly, in urine samples, unsupervised PCA was conducted for T_2-T_0 comparisons within each group (**Figure S1.3**). These analyses were used to evaluate within-group temporal variation and to visualize time-dependent differences in metabolic profiles without relying on supervised classification approaches.

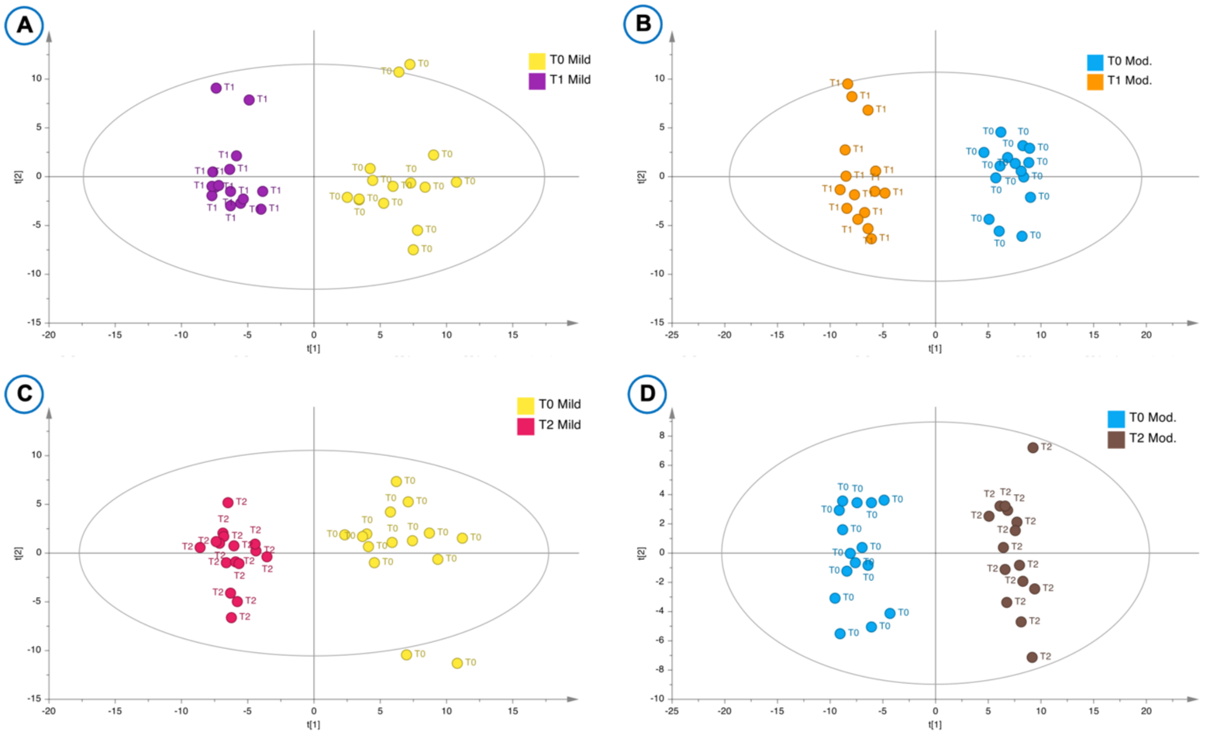

Figures S1.2 PCA score plots of plasma samples comparing different time points. (A) T_1-T_0 in the mild hypothermia group; (B) T_1-T_0 in the moderate hypothermia group; (C) T_2-T_0 in the mild hypothermia group; (D) T_2-T_0 in the moderate hypothermia group.

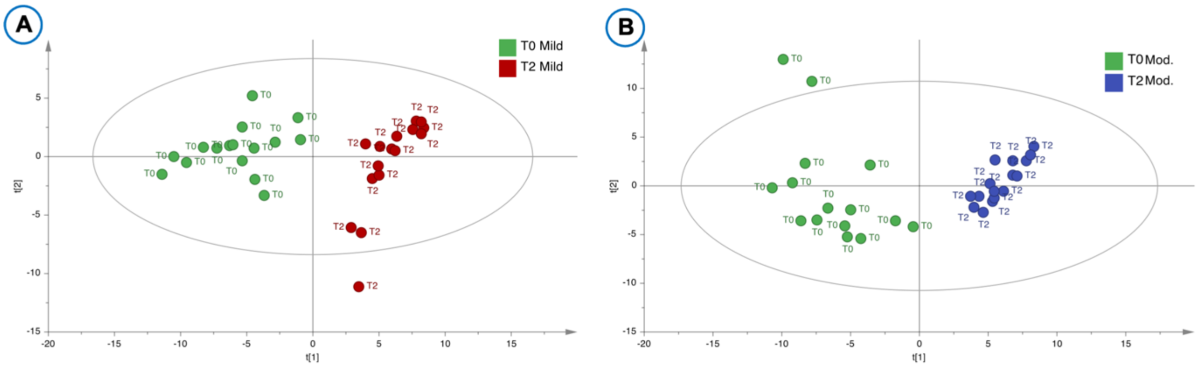

Figures S1.3 PCA score plots of urine samples comparing T_2-T_0. (A) Mild hypothermia group; (B) Moderate hypothermia group.

S1.2.2 Identification of Differential Metabolites Using Fold Change and Volcano Plot Analysis

Volcano plot analyses were performed using MetaboAnalyst 6.0 to identify significantly altered metabolites between post-CPB time points (T_1 and T_2) and the baseline (T_0) in both plasma and urine samples. In the volcano plot analysis, metabolites with a fold change (FC) > 2 and an FDR-adjusted p-value < 0.05 were considered significant. The resulting distributions are shown in Figure S1.4, and detailed results (including FC, $\log_2$(FC), p-values, and $-\log_{10}$(p)) are provided in Supplementary_File_S3_Volcano_Pathway.xlsx.

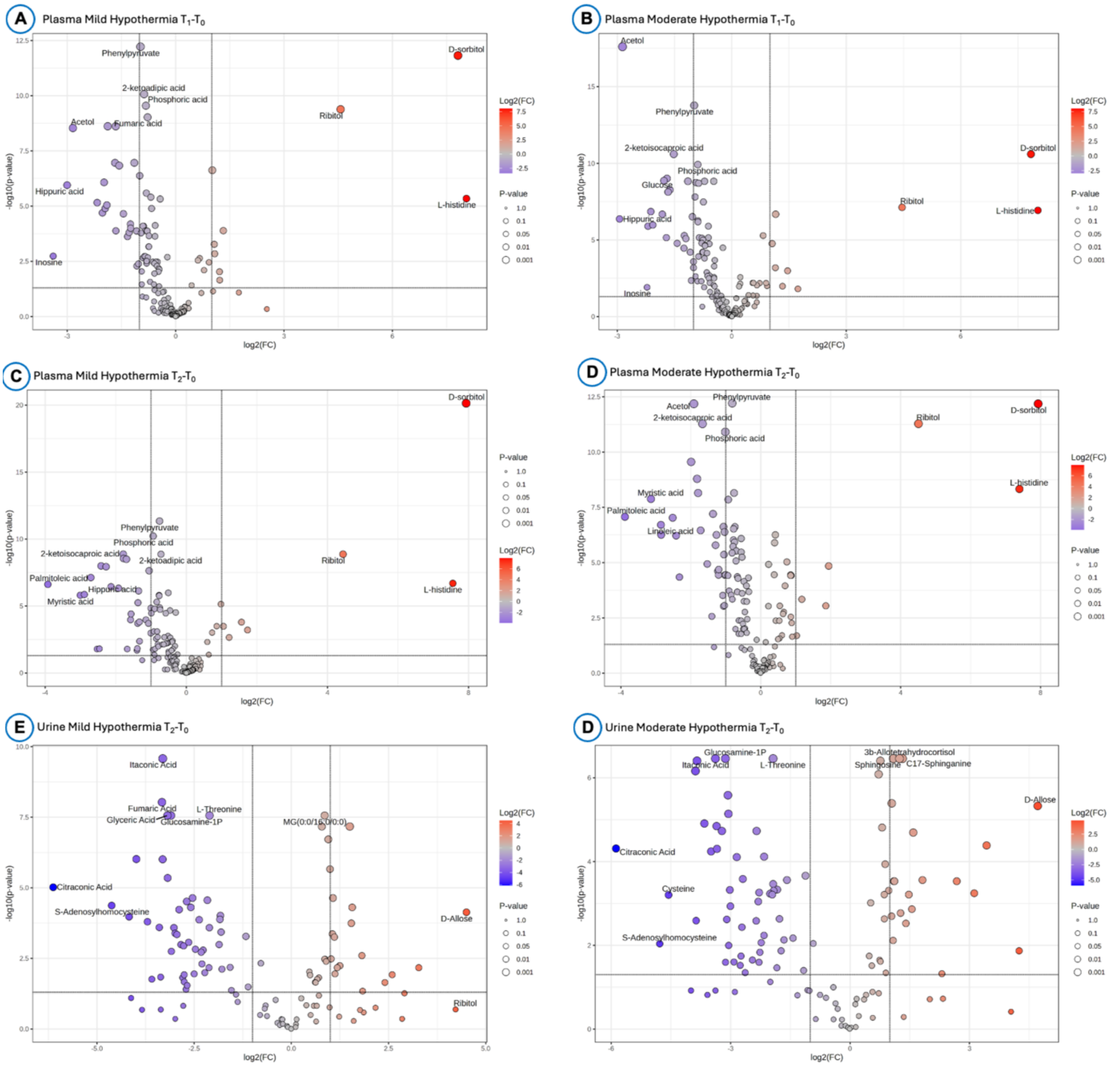

Figure S1.4 Volcano plots illustrating metabolite changes over time in mild and moderate hypothermia groups: (A) Plasma T_1-T_0 in the mild hypothermia group, (B) Plasma T_1-T_0 in the moderate hypothermia group, (C) Plasma T_2-T_0 in the mild hypothermia group, (D) Plasma T_2-T_0 in the moderate hypothermia group, (E) Urine T_2-T_0 in the mild hypothermia group, and (F) Urine T_2-T_0 in the moderate hypothermia group.

S.1.2.3 Pathway Analysis in Plasma and Urine Samples

Metabolite selection for pathway analysis was based on statistically significant metabolites (FC > 2, FDR-adjusted $p < 0.05$) identified in the volcano plot analysis. Pathway analysis was performed using MetaboAnalyst (version 6.0) and the KEGG database. The pathway impact and significance for each group and time point (T_1-T_0, T_2-T_0) are summarized in the Figure S1.5 below. Detailed results, including all pathway statistics for plasma and urine samples, are provided in Supplementary_File_S3_Volcano_Plots_and_Pathway_Analysis.xlsx.

Among plasma samples, pathway analysis for the mild hypothermia group at T_1-T_0 revealed significant enrichment in galactose metabolism and arginine biosynthesis, while the moderate hypothermia group at the same time point showed similar findings. At T_2-T_0, both mild and moderate hypothermia groups exhibited prominent alterations in valine, leucine and isoleucine biosynthesis, galactose metabolism, and unsaturated fatty acid biosynthesis, as well as notable changes in glycerolipid metabolism.

In urine samples, alanine, aspartate and glutamate metabolism, the citrate cycle (TCA cycle), glyoxylate and dicarboxylate metabolism, glycine, serine and threonine metabolism, and glycerolipid metabolism were among the most significantly affected pathways for both mild and moderate hypothermia groups at T_2-T_0. Notably, valine, leucine and isoleucine biosynthesis was also identified as a key pathway in both groups.

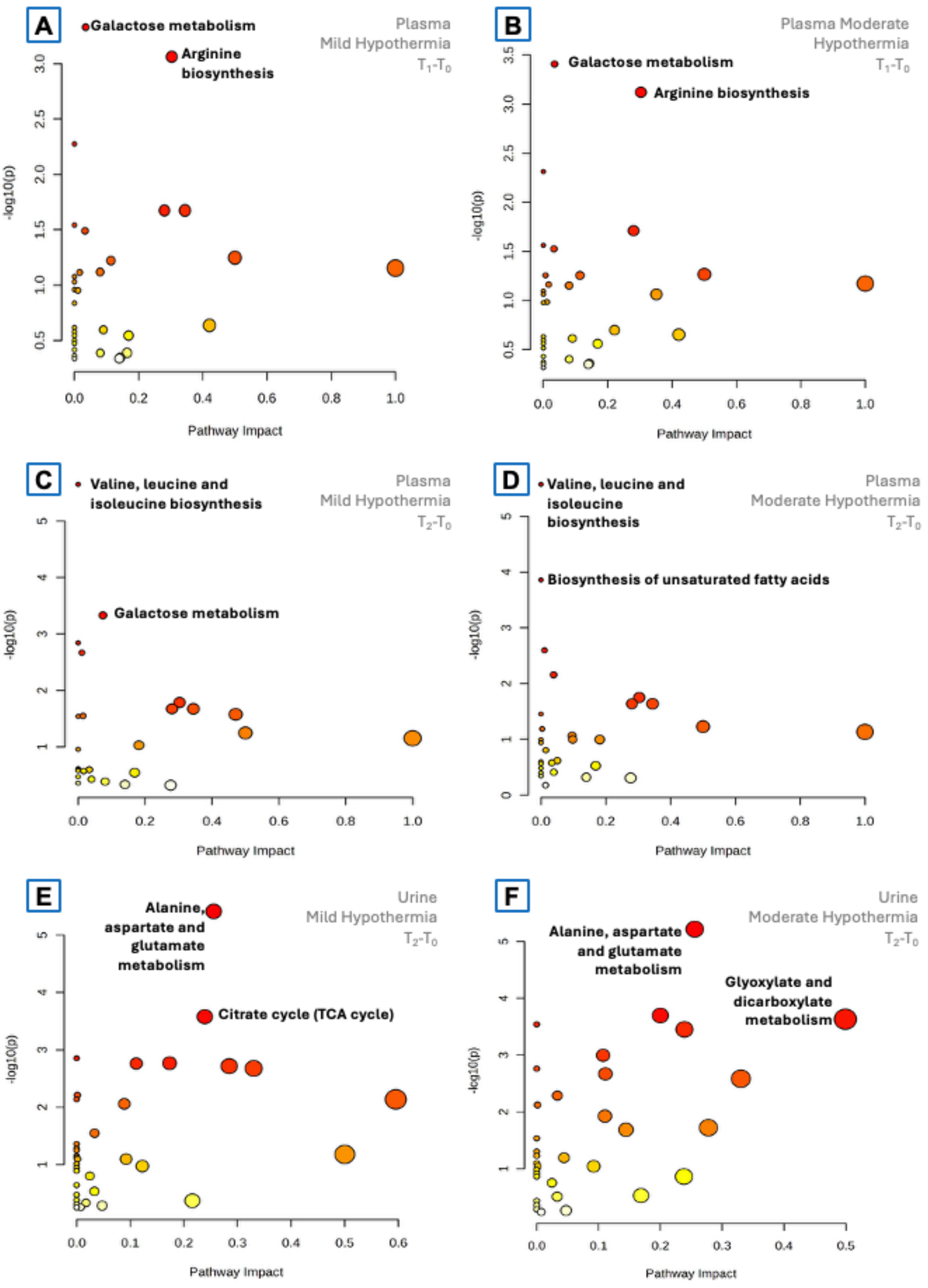

Figure S1.5 Pathway analysis results for plasma and urine samples in mild and moderate hypothermia groups: (A) Plasma T_1-T_0 in the mild hypothermia group, (B) Plasma T_1-T_0 in the moderate hypothermia group, (C) Plasma T_2-T_0 in the mild hypothermia group, (D) Plasma T_2-T_0 in the moderate hypothermia group, (E) Urine T_2-T_0 in the mild hypothermia group, and (F) Urine T_2-T_0 in the moderate hypothermia group.

S1.2.4 Statistical Analysis Results for Plasma and Urine Samples

The data matrices obtained from GC-MS and LC-qTOF-MS analyses were processed to ensure data consistency and accuracy across all samples. Each dataset was normalized to the total peak area to account for day-to-day variations resulting from multiple analytical batches. Missing values were imputed using half the value of the smallest concentration within the respective metabolite group.

The time points at which samples were collected (T_1–T_0 and T_2–T_0) were compared using the Wilcoxon test. Metabolites that did not show statistically significant changes between the time points were excluded from the analysis. In this way, only metabolites with significant changes were included in the evaluation.

Statistical analyses were performed using the Mann-Whitney U test to compare metabolite levels between mild and moderate hypothermia groups at different time points (T1-T0 and T2-T0). This non-parametric test was chosen to accommodate data distributions that may not conform to normality assumptions.

Table S1.4 and **Table S1.5** present the results of Mann-Whitney U test analyses for plasma samples at the T_1-T_0 and T_2-T_0 time points, respectively. Similarly, **Table S1.6** summarizes the results for urine samples at the T_2-T_0 time point.

Table S1.4 T_1-T_0 time points: Statistical analysis results of GC-MS and LC-qTOF-MS-based metabolomic analysis of plasma samples using the Mann-Whitney U test

Metabolite (T_1-T_0 Plasma)	Method	Moderate Hypothermia (n=14)				Mild Hypothermia (n=15)				P value
		Median	Mean	Min	Max	Median	Mean	Min	Max	
(9Z,12Z,15Z)-octadecatrien-1-ol	MS/MS (+)	0.155	0.128	-0.292	0.099	0.145	0.159	-0.692	0.271	0.694
(9Z,12Z)-octadecadien-1-ol	MS/MS (+)	-0.118	-0.101	-0.118	0.264	-0.063	-0.096	0.048	0.317	0.383
1-pentadecanoyl-2-hydroxy-sn-glycero-3-phosphocholine	MS/MS (+)	-0.532	-0.601	-0.966	-0.288	-0.595	-0.578	-1.353	-0.132	0.631
1-Hydroxy-2-(9Z,12Z,15Z-octadecatrienoyl)-sn-glycero-2-phosphocholine	MS/MS (-)	0.090	0.038	-0.262	0.259	0.103	0.042	-0.471	0.283	0.827
1,6-anhydroglucose	GC-MS	0.003	0.078	-0.619	0.997	0.158	0.553	-0.646	2.190	0.190
2-amino-1-phenylethanol	GC-MS	-1.057	-1.244	-2.567	-0.694	-0.884	-1.079	-2.558	-0..424	0.190
2-amino-3-phosphonopropionic acid	GC-MS	-0.217	-0.198	-0.994	0.550	-0.688	-0.558	-2.200	0.55	0.315
2-aminoadipic acid	GC-MS	-0.350	-0.372	-0.777	0.236	-0.679	-0.541	-0.850	-0.022	0.089
2-hydroxybutyric acid	GC-MS	-0.416	-0.511	-1.051	-0.221	-0.549	-0.743	-3.803	-0.148	0.541
2-ketoadipic acid	GC-MS	-0.609	-0.630	-1.053	-0.355	-0.593	-0.628	-0.938	-0.371	0.727
2-ketoisocaproic acid	GC-MS	-1.135	-1.206	-1.776	-0.736	-1.188	-1.237	-1.876	-0.659	0.570
3-aminoisobutyric acid	GC-MS	-0.063	-0.081	-0.572	447	-0.081	-0.161	-0.605	0.167	0.513
3-indolelactic acid	GC-MS	-0.549	-0.546	-1.263	0.055	-0.489	-0.555	-2.214	0.073	0.760
3-methyl-2-oxobutanoic acid	GC-MS	-0.595	-0.576	-1.450	0.573	-0.364	-0.234	-1.608	1.045	0.138
3-phosphoglycerate	GC-MS	-0.352	-0.305	-0.710	0.141	-0.332	-0.264	-0.593	0.362	0.965
5-aminovaleric acid	GC-MS	-0.590	-0.559	-1.085	-0.048	-0.602	-0.560	-1.001	0.089	0.861
9-Decenoylcarnitine	MS/MS (+)	-0.221	-0.230	-0.699	0.081	-0.102	-0.238	-2.542	0.541	0.432
9,12-Hexadecadienoylcarnitine	MS/MS (+)	0.275	0.331	-0.313	1.214	0.100	0.158	-0.929	1.646	0.116
Acetol	GC-MS	-1.893	-1.875	-2.328	-1.334	-1.684	-1.764	-2.889	-0.899	0.315
Alanine	GC-MS	-0.438	-0.521	-0.897	-0.238	-0.499	-0.515	-0.843	-0.119	0.930
Allo-inositol	GC-MS	-0.306	-0.300	-0.522	-0.033	-0.438	-0.446	-1.134	0.003	0.275
Arachidonic_acid	MS/MS (+)	-0.368	-0.381	-1.210	0.096	-0.345	-0.210	-0.683	1.346	0.694
Asparagine	GC-MS	0.465	0.486	0.229	0.789	0.350	0.392	-0.084	0.803	0.295
Aspartic acid	GC-MS	-0.067	0.017	-0.348	0.565	-0.203	-0.236	-1.134	0.386	0.206
C17-sphinganine	MS/MS (+)	-0.019	-0.130	-0.691	0.357	-0.030	-0.057	-0.743	0.557	0.930
Cer(d18:0/12:0)	MS/MS (+)	0.212	0.224	-0.195	1.129	0.118	-0.027	-1.199	0.546	0.222
Cer(d18:0/14:0)	MS/MS (+)	0.411	0.524	-0.083	1.799	0.161	0.223	-0.722	0.928	0.127
Cer(d18:0/16:0)	MS/MS (+)	0.280	0.509	-0.171	2.068	-0.002	0.017	-1.126	0.727	**0.021**
Cer(d18:0/18:0)	MS/MS (+)	0.310	0.268	-0.573	1.017	0.208	0.159	-0.521	-0.110	0.407
Cer(t18:0/16:0)	MS/MS (+)	0.266	0.311	-0.269	1.241	0.123	0.027	-1.142	0.625	0.176
Cholesterol	GC-MS	-0.666	-0.714	-1.021	-0.379	-0.727	-0.695	-1.132	0.476	0.600
Choline	MS/MS (+)	-0.035	-0.012	-0.283	0.241	-0.009	-0.001	-0.228	0.324	0.793
Citric acid	GC-MS	-0.586	-0.614	-0.966	-0.275	-0.662	-0.696	-1.225	-0.265	0.432
Citrulline	GC-MS	-1.270	-1.294	-1.898	-0.821	-1.232	-1.240	-2.019	-0.188	0.760

Metabolite (T_1-T_0 Plasma)	Method	Moderate Hypothermia (n=14) Median	Mean	Min	Max	Mild Hypothermia (n=15) Median	Mean	Min	Max	P value
Cortisol	MS/MS (+)	-0.094	-0.135	-0.550	0.267	-0.071	-0.084	-0.450	0.292	0.631
Creatinine	GC-MS	-0.483	-0.473	-2.012	0.550	-0.215	-0.425	-2.065	0.372	0.432
Cysteine	GC-MS	-0.672	-0.581	-0.997	0.188	-0.625	-0.688	-1.900	-0.112	1.000
Cystine	GC-MS	0.804	0.971	0.159	1.930	1.088	0.775	-2.092	2.928	0.600
Decanoylcarnitine	MS/MS (+)	-0.134	-0.201	-0.8343	0.214	-0.184	-0.508	-5.387	0.720	0.663
Docosahexaenoic acid	MS/MS (+)	-0.177	-0.183	-0.727	0.232	-0.179	-0.306	-1.653	0.274	0.663
Docosahexanoic acid	MS/MS (-)	-0.332	-0.369	-1.446	0.176	-0.300	-0.318	-1.146	0.558	0.793
Dodecanoylcarnitine	MS/MS (+)	-0.023	-0.002	-0.443	0.460	-0.086	-0.258	-2.531	0.292	0.407
Fumaric acid	GC-MS	-0.538	-0.535	-0.743	-0.312	-0.509	-0.538	-0.768	-0.246	0.861
Galactitol	GC-MS	0.519	0.425	-2.599	1.907	0.415	0.278	-2.994	1.414	0.458
Glucopyranose	GC-MS	0.420	0.415	-0.230	1.196	0.339	0.468	0.031	1.383	0.827
Glucose	GC-MS	-1.142	-1.116	-1.624	-0.566	-1.163	-1.203	-2.073	-0.434	0.631
Glucose-6-phosphoric acid	GC-MS	-0.535	-0.542	-1.247	-0.045	-0.451	-0.494	-0.981	0.019	0.793
Glutamic acid	GC-MS	-0.284	-0.297	-0.920	0.151	-0.273	-0.254	-0.810	0.208	0.793
Glutamine	GC-MS	0.802	0.711	0.237	1.137	0.599	0.640	0.371	1.251	0.359
Glyceraldehyde	GC-MS	-0.676	-0.740	-1.275	-0.505	-0.773	-0.750	-1.445	-0.164	0.694
Glyceric acid	GC-MS	-0.504	-0.527	-0.866	-0.263	-0.634	-0.587	-1.137	-0.080	0.663
Glycerol	GC-MS	-1.243	-1.211	-2.341	0.007	-1.020	-1.151	-3.122	-0.027	0.275
Glycerol 1-phosphate	GC-MS	-0.928	-1.170	-2.751	-0.534	-0.880	-1.280	-3.285	-0.294	0.930
Glycerol-3-phosphate	GC-MS	-0.592	-0.883	-2.247	-0.197	-0.714	-0.992	-2.741	-0.142	0.827
Glycine	GC-MS	-0.267	-0.318	-0.633	-0.085	-0.350	-0.389	-0.824	-0.024	0.315
Hexadecenal	MS/MS (+)	0.109	0.079	-0.198	0.215	0.162	0.156	-0.110	0.349	**0.036**
Hippuric acid	GC-MS	-1.840	-2.128	-5.201	-0.797	-2.094	-1.958	-3.739	-0.356	0.760
Histidine	GC-MS	1.761	1.993	1.077	3.686	1.333	1.612	0.035	3.412	0.176
Inosine	GC-MS	-0.574	-0.910	-2.391	0.130	-2.014	-2.415	-10.383	0.092	**0.029**
Isoleucine	GC-MS	-0.569	-0.579	-1.182	-0.154	-0.707	-0.738	-1.290	-0.133	0.337
Lactic acid	GC-MS	-0.751	-0.791	-1.436	-0.236	-0.752	-0.744	-1.409	-0.007	0.727
Leucine	GC-MS	-0.813	-0.853	-2.185	0.124	-1.110	-1.110	-2.255	0.032	0.190
Linoleic acid	GC-MS	-0.920	-0.957	-2.081	-0.139	-1.030	-0.971	-2.263	-0.040	0.861
Lysine	GC-MS	-0.586	-0.554	-1.084	-0.048	-0.598	-0.560	-0.993	0.091	0.793
LysoPC(0:0/18:0)	MS/MS (+)	-0.449	-0.462	-0.703	-0.243	-0.438	-0.410	-0.536	-0.228	0.315
LysoPC(16:0)	MS/MS (+)	-0.307	-0.315	-0.480	-0.151	-0.312	-0.287	-0.383	-0.120	0.541
LysoPC(16:1(9Z))	MS/MS (+)	-0.317	-0.330	-0.743	0.016	-0.238	-0.324	-0.807	0.093	1.000
LysoPC(17:0)	MS/MS (+)	-0.562	-0.570	-0.948	-0.281	-0.562	-0.537	-1.052	-0.266	0.600
LysoPC(18:1(9Z))	MS/MS (+)	-0.040	-0.036	-0.306	0.230	0.026	0.030	-0.260	0.350	0.383
LysoPC(18:2(9Z,12Z))	MS/MS (+)	-0.057	-0.064	-0.315	0.207	-0.034	-0.013	-0.255	0.334	0.485
LysoPC(18:3(6Z,9Z,12Z))	MS/MS (+)	-0.007	-0.007	-0.101	0.091	0.002	-0.040	-0.641	0.108	0.861
LysoPC(18:4(6Z,9Z,12Z,15Z))	MS/MS (+)	-0.321	-0.377	-0.898	-0.011	-0.232	-0.335	-1.131	0.217	0.570
LysoPC(20:1(11Z))	MS/MS (+)	-0.318	-0.338	-0.692	-0.071	-0.332	-0.358	-0.828	0.007	0.896

Metabolite (T1-T0 Plasma)	Method	Moderate Hypothermia (n=14) Median	Mean	Min	Max	Mild Hypothermia (n=15) Median	Mean	Min	Max	P value
LysoPC(20:2(11Z,14Z))	MS/MS (+)	-0.271	-0.245	-0.767	0.156	-0.237	-0.171	-0.505	0.349	0.631
LysoPC(20:3(5Z,8Z,11Z))	MS/MS (+)	-0.057	-0.036	-0.710	0.458	0.067	0.086	-0.358	0.925	0.458
LysoPC(20:4(5Z,8Z,11Z,14Z))	MS/MS (+)	-0.003	-0.042	-0.373	0.514	-0.035	-0.012	-0.292	0.331	0.760
LysoPC(20:5(5Z,8Z,11Z,14Z,17Z))	MS/MS (+)	0.043	0.038	-0.055	0.154	0.037	0.028	-0.377	0.175	0.663
LysoPC(22:5(4Z,7Z,10Z,13Z,16Z))	MS/MS (+)	-0.215	-0.216	-0.768	0.162	-0.121	-0.103	-0.557	0.513	0.383
LysoPC(P-18:0)	MS/MS (+)	-0.597	-0.627	-0.977	-0.348	-0.583	-0.619	-1.206	-1.386	0.600
LysoPE(0:0/16:0)	MS/MS (+)	-0.248	-0.236	-0.472	0.023	-0.135	-0.164	-0.486	0.025	0.138
LysoPE(18:2(9Z,12Z)/0:0)	MS/MS (+)	-0.011	0.001	-0.350	0.399	0.169	0.144	-0.324	0.518	0.176
LysoPE(22:6(4Z,7Z,10Z,13Z,16Z,19Z)/0:0)	MS/MS (+)	0.075	0.156	-0.283	1.028	0.319	0.168	-0.288	0.466	0.485
Malic acid	GC-MS	0.381	0.421	-0.185	1.025	0.285	0.324	-0.145	1.059	0.359
Malonic acid	GC-MS	0.313	0.388	-0.454	1.103	0.475	0.629	-0.015	1.824	0.163
Mannose	GC-MS	-0.953	-0.723	-1.418	0.753	-0.982	-0.937	-1.812	0.702	0.383
Methionine	GC-MS	-0.215	-0.227	-0.478	0.075	-0.351	-0.348	-0.746	0.234	0.150
MG(0:0/16:0/0:0)	MS/MS (+)	0.101	0.074	-0.327	0.185	0.182	0.138	-0.069	0.306	0.067
MG(0:0/20:5(5Z,8Z,11Z,14Z,17Z)/0:0)	MS/MS (+)	-0.265	-0.220	-0.849	1.102	-0.025	-0.297	-3.642	1.801	0.275
MG(18:0/0:0/0:0)	MS/MS (+)	0.136	0.143	0.013	0.289	0.166	0.176	-0.016	0.332	0.239
MG(18:1(9Z)/0:0/0:0)	MS/MS (+)	-0.576	-0.544	-1.023	-0.140	-0.223	-0.723	-4.717	0.260	**0.032**
Myristic acid	GC-MS	-1.927	-1.830	-3.004	-0.154	-1.457	-1.573	-3.634	-0.460	0.256
Myristoyl ethanolamide	MS/MS (+)	-0.028	-0.033	-0.708	0.502	0.031	-0.026	-0.376	0.285	0.965
N-ethylglycine	GC-MS	-0.365	-0.378	-1.384	0.365	-0.302	-0.251	-0.962	0.661	0.570
O-[(9Z)-17-carboxyheptadec-9-enoyl]carnitine	MS/MS (+)	0.384	0.581	-0.175	1.924	0.258	0.394	-0.150	1.361	0.359
O-phosphocolamine	GC-MS	-0.302	-0.179	-0.990	1.134	-0.099	-0.061	-0.641	0.598	0.275
Octanoylcarnitine	MS/MS (+)	-0.119	-0.143	-0.637	0.199	-0.163	-0.442	-5.124	0.699	0.600
Oleoylcarnitine	MS/MS (+)	-0.258	-0.289	-0.772	0.073	-0.329	-0.372	-0.955	0.042	0.295
Ornithine	GC-MS	-1.389	-1.413	-2.022	-0.698	-1.337	-1.508	-2.897	-0.741	0.930
Palmitic acid	MS/MS (-)	-0.478	-0.409	-0.932	0.282	-0.551	-0.441	-1.042	0.170	0.513
Palmitoleic acid	GC-MS	-1.776	-1.908	-4.577	-0.297	-1.916	-1.767	-3.417	-0.475	0.827
Palmitoylcarnitine	MS/MS (+)	-0.295	-0.379	-0.960	-0.080	-0.345	-0.376	-1.047	-0.047	1.000
PC(18:3(6Z,9Z,12Z)/22:6(4Z,7Z,10Z,13Z,16Z,19Z))	MS/MS (-)	0.200	0.204	-0.611	1.025	0.176	0.135	-0.875	0.672	0.760
PC(20:5(5Z,8Z,11Z,14Z,17Z)/22:6(4Z,7Z,10Z,13Z,16Z,19Z))	MS/MS (-)	0.380	0.402	-0.600	2.330	0.125	0.082	-0.758	0.983	0.150
Phenylalanine	GC-MS	-0.466	-0.466	-0.793	-0.077	-0.452	-0.464	-0.708	-0.247	0.861
Phenylpyruvate	GC-MS	-0.726	-0.719	-0.906	-0.474	-0.713	-0.685	-0.937	-0.344	0.570
Phosphocholine	MS/MS (+)	-0.158	-0.157	-0.371	0.060	-0.143	-0.153	-0.316	-0.019	0.793
Phosphoric acid	GC-MS	-0.654	-0.676	-0.892	-0.557	-0.638	-0.634	-0.930	-0.312	0.631
Pipecolic acid	GC-MS	-0.412	-0.645	-1.749	0.126	-0.456	-0.725	-1.664	0.058	0.694

Metabolite (T_1-T_0 Plasma)	Method	Moderate Hypothermia (n=14) Median	Mean	Min	Max	Mild Hypothermia (n=15) Median	Mean	Min	Max	P value
Proline	GC-MS	-0.343	-0.416	-1.019	-0.050	-0.580	-0.676	-1.542	-0.052	**0.049**
Putrescine	GC-MS	0.965	1.558	-0.270	4.916	0.377	1.005	-0.854	5.741	0.256
Pyroglutamic acid	GC-MS	-0.429	-0.446	-0.673	-0.111	-0.433	-0.420	-0.700	-0.159	0.663
Pyrophosphate	GC-MS	-0.165	-0.190	-0.869	0.254	-0.113	-0.104	-0.462	0.324	0.485
Pyruvic acid	GC-MS	-0.375	-0.408	-0.884	0.095	-0.378	-0.351	-0.967	0.652	0.930
Ribitol	GC-MS	1.466	1.403	0.359	2.131	1.639	1.538	0.559	2.402	0.407
Ribose-5-phosphate	GC-MS	-1.245	-1.269	-2.695	-0.681	-1.131	-1.296	-2.719	-0.353	0.861
Serine	GC-MS	-0.027	-0.043	-0.341	0.267	-0.096	-0.156	-0.724	0.206	0.485
SM(d18:1/14:0)	MS/MS (+)	0.142	0.267	-0.500	1.409	0.697	0.635	-0.225	1.416	0.163
Sorbitol	GC-MS	1.512	1.362	0.857	1.865	1.588	1.532	0.666	2.196	0.256
Sphingosine	MS/MS (+)	0.049	0.027	-0.277	0.484	-0.027	-0.067	-0.519	0.201	0.432
Stearic acid	MS/MS (+)	0.149	0.138	-0.200	0.301	0.159	0.164	-0.016	0.369	0.600
Stearoylethanolamide	MS/MS (+)	0.057	0.076	-0.183	0.409	-0.040	-0.000	-0.309	0.275	0.222
Sucrose	GC-MS	-0.497	-0.568	-1.818	-0.0009	-0.543	-0.747	-3.557	2.165	0.727
Threitol	GC-MS	-0.571	-0.597	-1.125	-0.393	-0.638	-0.655	-1.079	-0.255	0.407
Threo-β-hydroxyaspartic acid	GC-MS	0.481	0.581	-0.170	1.629	0.380	0.604	-0.069	2.033	0.631
Threonic acid	GC-MS	-0.366	-0.423	-0.804	-0.110	-0.370	-0.410	-0.792	-0.038	0.861
Threonine	GC-MS	-0.255	-0.222	-0.447	0.138	-0.242	-0.253	-0.864	0.320	0.631
Trans-4-hydroxyproline	GC-MS	-0.085	-0.131	-0.455	0.110	-0.077	-0.232	-2.021	0.083	0.965
Tryptophane	MS/MS (+)	0.701	0.671	0.118	1.412	0.683	0.828	0.269	2.857	0.694
Tyrosine	GC-MS	-1.154	-1.238	-1.881	-0.615	-1.180	-1.144	-2.094	-0.326	0.663
Uric acid	GC-MS	0.288	0.283	-0.667	1.343	0.051	-0.075	-2.482	2.282	0.239
Urocanic acid	GC-MS	0.522	0.434	-0.792	1.152	0.457	0.476	-1.007	1.819	0.930
Valine	GC-MS	-0.630	-0.627	-1.160	-0.221	-0.665	-0.669	-1.947	0.906	0.458
Xylitol	GC-MS	-0.419	-0.466	-0.950	-0.157	-0.503	-0.535	-0.775	-0.282	0.222
α-ketoglutaric acid	GC-MS	-0.532	-0.627	-1.886	0.537	-0.611	-0.572	-1.872	1.176	0.793
β-alanine	GC-MS	-0.658	-0.672	-1.185	-0.273	-0.492	-0.553	-1.103	-0.211	0.176
β-glycerolphosphate	GC-MS	-0.661	-0.838	-1.899	-0.368	-0.696	-0.940	-2.426	-0.228	0.827
γ-linolenic acid	MS/MS (+)	0.888	0.828	-0.310	1.827	0.813	0.992	-0.052	2.731	0.663

min: minimum, max: maximum, bold P-values indicate statistical significance. Cer: ceramide, MG: monoglyceride, PC: phosphatidylcholine, SM: sphingomyelin, LysoPC: lysophosphatidylcholine, LysoPE: lysophosphatidylethanolamine.

Table S1.5 T_2-T_0 time points: Statistical analysis results of GC-MS and LC-qTOF-MS-based metabolomic analysis of plasma samples using the Mann-Whitney U test

		**Moderate Hypothermia (n=14)**				**Mild Hypothermia (n=15)**				**P**
**Metabolite** (T_2-T_0 Plasma)	**Method**	**Median**	**Mean**	**Min**	**Max**	**Median**	**Mean**	**Min**	**Max**	**value**
(9Z,12Z,15Z)-octadecatrien-1-ol	MS/MS (+)	0.279	0.255	-0.665	-0.169	0.210	0.152	-1.987	-0.148	0.275
(9Z,12Z)-octadecadien-1-ol	MS/MS (+)	-0.423	-0.419	-0.058	0.458	-0.506	-0.650	-0.780	0.453	0.275
1-pentadecanoyl-2-hydroxy-sn-glycero-3-phosphocholine	MS/MS (+)	-0.401	-0.372	-0.699	-0.004	-0.280	-0.311	-0.746	-0.003	0.458
1,6-anhydroglucose	GC-MS	1.079	1.305	-0.391	3.251	1.130	1.226	-0.728	3.263	1.000
2-amino-1-phenylethanol	GC-MS	-0.466	-0.572	-2.023	0.833	-0.281	-0.321	-2.076	1.671	0.359
2-amino-3-phosphonopropionic acid	GC-MS	-0.171	-0.037	-0.617	0.751	-0.400	-0.386	-1.666	1.114	0.067
2-aminoadipic acid	GC-MS	-0.120	-0.172	-1.413	0.654	-0.458	-0.460	-1.151	0.430	0.239
2-hydroxybutyric acid	GC-MS	-0.471	-0.481	-0.896	-0.124	-0.516	-0.712	-2.558	0.082	0.570
2-ketoadipic acid	GC-MS	-0.578	-0.573	-0.987	-0.229	-0.487	-0.542	-0.870	-0.249	0.827
2-ketoisocaproic acid	GC-MS	-1.268	-1.260	-1.808	-0.909	-1.196	-1.286	-2.712	-0.641	0.861
3-aminoisobutyric acid	GC-MS	-0.055	-0.075	-0.953	0.741	-0.192	-0.211	-0.583	0.145	0.089
3-phosphoglycerate	GC-MS	0.495	0.513	-0.143	1.456	0.173	0.265	-0.418	1.306	0.190
5-aminovaleric acid	GC-MS	-0.531	-0.508	-1.044	-0.002	-0.567	-0.490	-1.239	-0.075	1.000
9-decenoylcarnitine	MS/MS (+)	0.269	0.185	-0.294	0.505	0.124	-0.107	-3.424	0.426	0.295
9,12-hexadecadienoylcarnitine	MS/MS (+)	0.588	0.531	-0.256	2.097	0.225	0.121	-611	1.201	0.127
Acetol	GC-MS	-1.697	-1.600	-2.106	-0.452	-1.665	-1.666	-2.788	-0.696	0.861
Alanine	GC-MS	-0.336	-0.400	-0.960	0.077	-0.270	-0.296	-0.827	0.078	0.275
Allo-inositol	GC-MS	-0.071	-0.069	-0.422	0.193	-0.239	-0.255	-1.249	0.330	0.222
Arachidonic acid	GC-MS	-0.797	-0.786	-1.593	-0.161	-0.780	-0.882	-2.530	-0.321	0.861
Asparagine	GC-MS	0.483	0.462	-0.085	0.787	0.328	0.375	-0.225	0.905	0.275
Aspartic acid	GC-MS	-0.284	-0.142	-0.577	1.439	-0.312	-0.362	-1.533	0.011	0.861
Cer(d18:0/12:0)	MS/MS (+)	0.249	0.290	-0.054	0.793	0.189	0.121	-1.122	0.688	0.359
Cer(d18:0/14:0)	MS/MS (+)	0.430	0.464	-0.003	1.046	0.363	0.260	-0.984	1.138	0.127
Cer(d18:0/16:0)	MS/MS (+)	0.379	0.457	-0.134	1.261	0.129	0.087	-1.133	0.884	**0.040**
Cer(d18:0/18:0)	MS/MS (+)	0.324	0.381	-0.604	1.026	0.276	0.116	-0.968	0.707	0.222
Cer(t18:0/16:0)	MS/MS (+)	0.344	0.378	-0.145	0.926	0.165	0.116	-1.281	0.707	**0.045**
Cholesterol	GC-MS	-0.596	-0.602	-0.968	-0.222	-0.590	-0.567	-1.038	0.400	0.965
Choline	MS/MS (+)	-0.277	-0.223	-0.430	0.124	-0.216	-0.207	-0.384	0.014	0.631
cis-5-Tetradecenoylcarnitine	MS/MS (+)	0.332	0.313	-0.273	1.114	0.121	0.143	-0.704	1.199	0.337
Citric acid	GC-MS	-0.463	0.661	-0.935	15.639	-0.521	-0.561	-0.986	-0.205	0.359
Citrulline	GC-MS	-1.262	-1.360	-2.289	-0.874	-1.277	-1.329	-2.106	-0.512	0.896
Creatinine	GC-MS	-0.593	-0.571	-1.636	0.272	-0.223	-0.527	-1.760	0.125	0.631
Cysteine	GC-MS	-0.746	-0.705	-1.135	0.0003	-0.670	-0.745	-1.919	-0.237	0.727
Cystine	GC-MS	0.388	0.585	-0.010	1.793	0.157	0.174	-2.176	1.882	0.097
Docosahexaenoic.acid	MS/MS (+)	-0.481	-0.563	-1.475	-0.034	-0.498	-0.914	-5.973	0.175	0.727

Metabolite (T_2-T_0 Plasma)	Method	Moderate Hypothermia (n=14) Median	Mean	Min	Max	Mild Hypothermia (n=15) Median	Mean	Min	Max	P value
Docosahexanoic.acid	MS/MS (-)	-0.635	-0.803	-2.327	-0.128	-0.650	-0.922	-3.166	-0.060	0.965
Dodecanoylcarnitine	MS/MS (+)	0.281	0.304	-0.258	1.052	0.130	0.042	-2.524	1.023	0.315
Fumaric.acid	GC-MS	-0.331	-0.318	-0.551	-0.029	-0.409	-0.385	-0.672	-0.095	0.315
Glucose	GC-MS	-0.795	-0.781	-1.172	-0.245	-0.856	-0.857	-1.364	-0.175	0.513
Glucose-6-phosphoric acid	GC-MS	-0.504	-0.468	-1.077	0.073	-0.215	-0.315	-0.975	0.024	0.150
Glutamic acid	GC-MS	-0.198	-0.182	-0.713	0.629	-0.187	-0.207	-1.239	0.747	0.861
Glutamine	GC-MS	0.510	0.478	-0.075	0.929	0.504	0.511	-0.116	0.958	0.930
Glyceraldehyde	GC-MS	-0.524	-0.557	-0.919	-0.263	-0.604	-0.623	-1.673	0.111	0.930
Glyceric acid	GC-MS	-0.566	-0.540	-0.914	-0.146	-0.707	-0.620	-1.308	-0.089	0.315
Glycerol	GC-MS	-1.524	-1.552	-2.717	-0.238	-1.583	-1.576	-3.108	-769	0.930
Glycerol 1-phosphate	MS/MS (+)	-0.892	-1.092	-2.404	-0.127	-0.894	-1.249	-3.366	-0.224	0.861
Glycerol-3-phosphate	GC-MS	-0.732	-0.850	-2.012	-0.017	-0.649	-0.991	-2.745	-0.145	0.965
Glycine	GC-MS	-0.255	-0.277	-0.611	0.008	-0.349	-0.327	-0.742	0.087	0.631
Hexadecenal	MS/MS (+)	0.069	0.053	-0.140	0.171	0.067	0.070	-0.129	0.178	0.485
Hippuric.acid	GC-MS	-1.849	-2.083	-5.059	-0.477	-2.081	-1.935	-3.795	-0.130	0.896
Histidine	GC-MS	1.145	1.255	0.484	2.114	1.011	1.143	0.548	2.715	0.337
Inosine	GC-MS	-0.221	-0.699	-2.380	1.539	-1.246	-1.834	-9.220	0.160	0.127
Isoleucine	GC-MS	-0.108	-0.144	-0.462	0.068	-0.205	-0.199	-0.398	0.034	0.222
Lactic acid	GC-MS	-0.484	-0.406	-0.745	0.233	-0.323	-0.369	-1.398	0.416	0.570
Leucine	GC-MS	-1.151	-1.178	-1.742	-0.226	-1.121	-1.291	-2.317	-0.212	0.793
Linoleic acid	GC-MS	-1.545	-1.586	-2.769	-0.509	-1.620	-1.560	-2.904	-0.514	1.000
Lysine	GC-MS	-0.527	-0.504	-1.031	0.0002	-0.558	-0.486	-1.231	-0.074	1.000
LysoPC(0:0/18:0)	MS/MS (+)	-0.251	-0.260	-0.647	-0.017	-0.189	-0.198	-0.436	0.060	0.458
LysoPC(16:1(9Z))	MS/MS (+)	-0.390	-0.339	-0.576	0.070	-0.246	-0.317	-0.687	0.011	0.793
LysoPC(17:0)	MS/MS (+)	-0.369	-0.350	-0.646	0.001	-0.267	-0.265	-0.555	0.002	0.206
LysoPC(18:1(9Z))	MS/MS (+)	-0.288	-0.256	-0.442	0.032	-0.224	-0.222	-0.458	0.047	0.432
LysoPC(18:2(9Z,12Z))	MS/MS (+)	-0.391	-0.352	-0.617	0.100	-0.331	-0.320	-0.586	-0.081	0.383
LysoPC(18:3(6Z,9Z,12Z))	MS/MS (+)	0.159	0.158	0.030	0.292	0.141	0.110	-0.477	0.272	0.600
LysoPC(18:4(6Z,9Z,12Z,15Z))	MS/MS (+)	-0.445	-0.403	-0.696	0.085	-0.284	-0.362	-1.093	-0.006	0.383
LysoPC(20:1(11Z))	MS/MS (+)	-0.289	-0.319	-0.689	-0.011	-0.256	-0.334	-0.772	0.058	0.965
LysoPC(20:2(11Z,14Z))	MS/MS (+)	-0.493	-0.573	-1.093	0.083	-0.536	-0.538	-0.857	-0.158	0.827
LysoPC(20:3(5Z,8Z,11Z))	MS/MS (+)	-0.566	-0.637	-1.363	0.168	-0.470	-0.579	-1.093	-0.160	0.570
LysoPC(20:4(5Z,8Z,11Z,14Z))	MS/MS (+)	-0.368	-0.306	-0.586	0.387	-0.285	-0.265	-0.601	0.022	0.337
LysoPC(20:5(5Z,8Z,11Z,14Z,17Z))	MS/MS (+)	0.078	0.096	0.007	0.192	0.097	0.081	-0.321	0.214	0.600
LysoPC(22:5(4Z,7Z,10Z,13Z,16Z))	MS/MS (+)	-0.473	-0.533	-1.170	0.135	-0.487	-0.514	-1.087	-0.144	0.694
LysoPC(P-18:0)	MS/MS (+)	-0.445	-0.406	-0.755	-0.093	-0.360	-0.336	-0.618	0.021	0.337
LysoPE(18:2(9Z,12Z)/0:0)	MS/MS (+)	-0.543	-0.596	-1.015	0.153	-0.559	-0.601	-1.019	-0.006	0.861
LysoPE(22:6(4Z,7Z,10Z,13Z,16Z,19Z)/0:0)	MS/MS (+)	-0.639	-0.602	-1.047	0.415	-0.591	-0.761	-2.276	0.047	0.896

Metabolite (T_2-T_0 Plasma)	Method	Moderate Hypothermia (n=14) Median	Mean	Min	Max	Mild Hypothermia (n=15) Median	Mean	Min	Max	P value
Malic acid	GC-MS	0.363	0.371	-0.322	1.374	0.135	0.145	-0.772	0.956	0.295
Malonic acid	GC-MS	0.479	0.596	-0.353	2.451	0.808	0.604	-0.109	1.154	0.570
Mannose	GC-MS	-0.778	-0.446	-1.205	1.294	-0.887	-0.711	-1.475	0.661	0.239
Methionine	GC-MS	-0.574	-0.548	-0.960	-0.143	-0.587	-0.602	-1.070	-0.193	0.513
MG(0:0/16:0/0:0)	MS/MS (+)	0.085	0.051	-0.208	0.246	0.050	0.047	-0.126	0.156	0.827
MG(0:0/20:5(5Z,8Z,11Z,14Z,17Z)/0:0)	MS/MS (+)	-0.756	-0.952	-2.408	-0.351	-1.014	-1.492	-6.578	0.239	0.760
MG(18:0/0:0/0:0)	MS/MS (+)	0.269	0.265	-0.009	0.440	0.234	0.192	-0.800	0.568	0.432
MG(18:1(9Z)/0:0/0:0)	MS/MS (+)	-0.930	-1.101	-2.589	-0.555	-0.906	-1.692	-7.743	0.011	1.000
Myristic acid	GC-MS	-2.094	-2.092	-3.190	-0.387	-1.748	-1.825	-3.756	-0.728	0.275
O-[(9Z)-17-carboxyheptadec-9-enoyl]carnitine	MS/MS (+)	1.041	1.086	0.285	2.957	0.662	0.815	0.092	2.240	0.239
O-phosphocolamine	GC-MS	-0.371	-0.355	-1.306	0.802	-0.205	-0.294	-0.906	0.216	0.407
Oleoylcarnitine	MS/MS (+)	-0.149	-0.127	-0.313	0.074	-0.210	-0.198	-0.818	0.225	0.383
Ornithine	GC-MS	-1.529	-1.487	-2.070	-0.834	-1.347	-1.559	-2.899	-0.715	0.793
Palmitic acid	MS/MS (-)	-1.099	-1.054	-1.491	-0.450	-1.034	-1.041	-1.510	-0.617	0.631
Palmitoleic acid	GC-MS	-2.114	-2.255	-5.223	-0.576	-2.319	-2.121	-3.674	-0.526	0.965
PC(18:3(6Z,9Z,12Z)/22:6(4Z,7Z,10Z,13Z,16Z,19Z))	MS/MS (-)	0.632	0.656	0.010	1.475	0.446	0.469	-0.314	1.144	0.239
PC(20:5(5Z,8Z,11Z,14Z,17Z)/22:6(4Z,7Z,10Z,13Z,16Z,19Z))	MS/MS (-)	0.506	0.586	-0.536	2.310	0.382	0.488	-0.369	1.701	0.793
Phenylalanine	GC-MS	-0.531	-0.525	-0.856	-0.152	-0.521	-0.508	-0.843	-0.179	0.827
Phenylpyruvate	GC-MS	-0.680	-0.628	-0.873	-0.283	-0.560	-0.572	-0.834	-0.397	0.315
Phosphoric acid	GC-MS	-0.730	-0.719	-1.035	-0.310	-0.726	-0.698	-0.860	-0.366	0.930
Pipecolic acid	GC-MS	-0.422	-0.597	-1.659	0.230	-0.485	-0.722	-1.596	-0.032	0.570
Proline	GC-MS	-0.590	-0.654	-1.325	-0.230	-0.685	-0.831	-1.582	-0.377	0.222
Pyroglutamic acid	GC-MS	-0.466	-0.414	-0.575	-0.195	-0.402	-0.431	-0.869	-0.093	0.663
Pyrophosphate	GC-MS	-0.478	-0.469	-1.090	0.295	-0.293	-0.342	-1.216	0.252	0.239
Pyruvic acid	GC-MS	0.180	0.168	-0.383	0.616	0.073	0.205	-0.686	1.010	0.930
Ribitol	GC-MS	1.479	1.360	0.688	1.825	1.585	1.407	0.412	2.027	0.458
Ribose-5-phosphate	GC-MS	-0.997	-1.030	-2.483	0.152	-1.011	-1.172	-2.450	-0.520	0.760
Serine	GC-MS	-0.254	-0.194	-0.633	0.277	-0.345	-0.362	-0.852	0.170	0.206
Sorbitol	GC-MS	1.598	1.472	0.596	1.875	1.685	1.665	1.314	2.010	0.127
Sphingosine	MS/MS (+)	0.087	0.071	-0.130	0.249	0.081	0.034	-0.448	0.296	0.930
Stearic acid	MS/MS (+)	0.291	0.249	-0.274	0.514	0.186	0.196	-0.552	0.589	0.295
Stearoylethanolamide	MS/MS (+)	0.130	0.153	0.047	0.327	0.113	0.057	-0.391	0.255	0.190
Sucrose	GC-MS	-0.530	-0.601	-1.725	-0.103	-0.571	-1.070	-3.464	-0.239	0.760
Threitol	GC-MS	-0.523	-0.541	-1.017	-0.351	-0.583	-0.639	-1.301	-0.225	0.337
Threonic acid	GC-MS	-0.292	-0.299	-0.792	0.077	-0.215	-0.275	-0.690	-0.014	0.663
Threonine	GC-MS	-0.441	-0.394	-0.742	0.068	-0.431	-0.475	-1.073	0.104	0.600

Metabolite (T_2-T_0 Plasma)	Method	Moderate Hypothermia (n=14) Median	Mean	Min	Max	Mild Hypothermia (n=15) Median	Mean	Min	Max	P value
Trans-4-hydroxyproline	GC-MS	-0.157	-0.141	-0.415	0.075	-0.072	-0.236	-2.573	0.145	0.513
Tryptophane	MS/MS (+)	0.510	0.508	0.032	1.053	0.476	0.540	0.191	1.204	0.930
Tyrosine	GC-MS	-1.017	-1.116	-1.794	-0.543	-1.022	-1.013	-2.166	-0.332	0.485
Urocanic acid	GC-MS	0.740	0.572	-0.614	1.207	0.717	0.706	-0.124	2.094	0.965
Valine	GC-MS	0.286	0.278	-1.025	1.478	0.175	0.340	-0.852	1.823	0.663
Xylitol	GC-MS	-0.174	-0.115	-0.646	0.609	-0.430	-0.376	-0.688	0.069	0.061
α-ketoglutaric.acid	GC-MS	-0.489	-0.597	-1.623	-0.240	-0.533	-0.636	-2.166	-0.047	0.570
β-alanine	GC-MS	-0.525	-0.509	-0.947	0.083	-0.338	-0.415	-0.877	-0.212	0.256
β-glycerolphosphate	GC-MS	-0.544	-0.689	-1.589	-0.117	-0.737	-0.890	-2.343	-0.187	0.600
γ-Linolenic acid	MS/MS (+)	-0.342	-0.372	-0.787	0.029	-0.504	-0.435	-1.365	0.739	0.432

min: minimum, max: maximum, bold p-values indicate statistical significance. Cer: ceramide, MG: monoglyceride, PC: phosphatidylcholine, LysoPC: lysophosphatidylcholine, LysoPE: lysophosphatidylethanolamine.

Table S1.6 T_2-T_0 time points: Statistical analysis results of GC-MS and LC-qTOF-MS-based metabolomic analysis of urine samples using the Mann-Whitney U test

		**Moderate Hypothermia (n=14)**				**Mild Hypothermia (n=15)**				**P**
**Metabolite** (T_2-T_0 Urine)	**Method**	**Median**	**Mean**	**Min**	**Max**	**Median**	**Mean**	**Min**	**Max**	**value**
(9E)-9-nitrooctadecenoic acid	MS/MS (+)	-0.573	-1.525	-7.352	0.017	-0.610	-1.470	-8.545	-0.127	0.694
2-(2-phenylacetoxy)propionylglycine	MS/MS (-)	-1.173	-1.177	-2.971	-0.105	-1.132	-1.299	-2.731	-0.200	0.965
2-amino-1-phenylethanol	GC-MS	-0.475	-0.442	-0.834	0.024	-0.526	-0.495	-1.488	0.671	0.513
2-hydroxybutyric acid	GC-MS	-0.472	-0.832	-4.111	0.192	-1.394	-2.004	-5.465	-0.087	**0.015**
2-keto-gulonic acid	GC-MS	-0.349	-1.082	-4.253	-0.099	-1.342	-2.332	-12.115	-0.325	0.055
2-methylbutyroylcarnitine	MS/MS (+)	-1.139	-2.650	-23.429	-0.060	-0.737	-0.840	-1.778	-0.257	0.239
2-octenoylcarnitine	MS/MS (+)	-0.595	-1.347	-8.950	1.162	-0.727	-0.957	-4.395	-0.040	0.663
2,3-diaminopropionic acid	MS/MS (+)	0.354	0.872	0.020	2.472	0.844	1.053	-0.231	2.871	0.359
2,3-dihydroxypropyl hexadecanoate	MS/MS (+)	0.786	0.971	0.236	2.160	0.674	0.718	-0.206	2.812	0.337
2,4-diaminobutyric acid	MS/MS (+)	1.106	1.017	-0.541	2.326	0.769	0.693	-1.315	2.502	0.359
3-aminoisobutanoic acid	GC-MS	-1.534	-1.470	-2.855	-0.075	-1.773	-1.782	-4.092	-358	0.383
3-hydroxypropanoic acid	GC-MS	0.177	0.124	-0.566	0.604	0.134	0.088	-0.767	0.722	0.694
3-methyl-2-oxobutanoic acid	GC-MS	-0.408	-0.903	-3.973	0.284	-1.352	-1.981	-5.301	-0.182	**0.029**
3-phosphoglyceric acid	GC-MS	1.467	1.602	0.116	4.192	1.103	1.552	0.478	7ç792	0.256
3β-allotetrahydrocortisol	MS/MS (+)	0.848	0.802	0.319	1.499	0.800	0.923	0.472	1.786	0.513
5-methylfuran-2-carboxylic acid	MS/MS (-)	-0.421	-2.028	-15.633	-0.050	-0.508	-0.918	-4.669	-0.030	0.896
5,6-dihydroxyeicosatrienoic acid	MS/MS (+)	0.499	0.529	0.041	1.393	0.624	0.654	-0.020	1.356	0.485
6-keto-decanoylcarnitine	MS/MS (+)	-0.909	-1.231	-5.710	-0.388	-1.269	-1.840	-5.379	-0.152	0.827
7a,12a-Dihydroxy-3-oxo-4-cholenoic acid	MS/MS (+)	0.275	0.412	-0.531	1.405	0.591	0.784	-3.748	5.395	0.694
9-decenoylcarnitine	MS/MS (+)	-1.241	-1.485	-3.500	-0.177	-1.573	-1.527	-3.533	-0.197	0.600
9-hydroxyoctadecanoic acid	MS/MS (-)	0.498	0.396	-1.084	1.883	0.410	0.500	-0.966	2.153	0.631
Acetoacetate	GC-MS	-0.515	-0.670	-3.074	1.023	-0.950	-1.136	-3.265	1.727	0.190
Acetylserine	GC-MS	-0.939	-1.171	-2.682	-0.336	-1.587	-1.813	-4.204	-0.355	**0.049**
Aspartyl-4-phosphate	MS/MS (-)	-1.122	-1.406	-4.075	-0.496	-1.078	-1.448	-3.992	-0.003	0.965
Aspartylphenylalanine	MS/MS (-)	-0.410	-2.083	-15.569	-0.010	-0.390	-1.130	-8.773	0.0009	0.570
C17-sphinganine	MS/MS (+)	0.936	0.887	0.384	1.390	0.656	0.583	-0.455	1.896	**0.021**
Cer(d18:0/14:0)	MS/MS (+)	0.561	0.561	0.094	1.029	0.626	0.586	-0.037	1.248	0.930
Cer(d18:0/18:0)	MS/MS (+)	0.907	0.772	0.023	1.487	0.829	0.744	0.002	1.728	0.965
Cer(d18:0/20:0)	MS/MS (+)	1.081	1.126	0.038	2.644	1.037	1.113	0.013	3.141	0.663
Cer(d18:1/22:0)	MS/MS (+)	0.599	0.739	0.106	1.398	0.727	0.762	0.005	1.785	0.965
Cer(d20:0/16:0(2OH))	MS/MS (+)	0.598	0.645	0.037	1.167	0.729	0.639	0.0001	1.448	0.965
Cer(t18:0/16:0)	MS/MS (+)	0.647	0.664	0.153	1.146	0.716	0.694	-0.101	1.941	0.896
Citraconic acid	GC-MS	-1.811	-1.879	-4.335	-0.454	-2.195	-2.042	-4.576	-0.0002	0.663
Citramalic acid	GC-MS	-0.714	-1.391	-4.247	-0.345	-1.089	-1.383	-5.864	2.113	0.965
Creatinine	MS/MS (+)	0.900	0.824	-1.267	2.181	0.863	1.065	-0.260	2.128	0.600
Cysteine	GC-MS	-1.748	-1.915	-5.112	0.165	-1.899	-1.896	-4.346	-0.052	0.861

Metabolite (T_2-T_0 Urine)	Method	Moderate Hypothermia (n=14) Median	Mean	Min	Max	Mild Hypothermia (n=15) Median	Mean	Min	Max	P value
Decanoylcarnitine	MS/MS (+)	-0.522	-1.303	-7.706	-0.011	-0.871	-1.363	-8.114	0.114	0.663
Dihydroxyacetone	GC-MS	-0.895	-1.123	-2.573	0.545	-0.998	-1.507	-7.043	0.176	0.600
Fumaric acid	GC-MS	-1.501	-1.685	-4.217	-0.463	-1.540	-1.550	-2.604	-0.594	0.827
Galactose	GC-MS	0.302	0.361	-0.097	1.063	0.357	0.334	-0.673	1.611	0.965
Glucoheptonic acid	GC-MS	-0.058	1.065	-1.218	15.833	-0.038	0.668	-0.475	6.126	0.827
Gluconic acid	GC-MS	-1.112	-0.866	-2.003	1.127	-0.823	-0.554	-2.467	1.479	0.359
Glucosamine-1P	GC-MS	-1.262	-1.483	-2.943	-0.593	-1.370	-1.616	-3.385	-0.813	0.359
Glucuronic acid	GC-MS	0.194	0.148	-0.631	0.670	0.157	0.171	-0.358	0.744	0.965
Glutamine	MS/MS (+)	-0.974	-1.210	-2.346	-0.165	-1.111	-1.093	-2.269	-0.130	0.760
Glyceraldehyde	GC-MS	-1.501	-1.686	-3.303	0.351	-1.540	-1.551	-4.705	-0.012	0.827
Glyceric acid	GC-MS	-1.364	-1.370	-2.533	0.094	-1.728	-1.779	-3.501	-0.744	0.206
Glycerol	GC-MS	-1.890	-1.919	-3.543	-0.699	-2.022	-1.828	-3.820	-0.201	0.694
Glycerol 1-phosphate	GC-MS	-0.081	0.520	-1.219	7.171	-0.037	1.279	-0.037	-2.912	0.861
Glycine	GC-MS	-0.562	-1.131	-3.758	-0.010	-0.671	-1.155	-3.309	0.006	0.663
Glycocholic acid	MS/MS (+)	0.548	0.581	-0.736	2.570	0.279	0.407	-0.574	1.671	0.600
Heptanoylcarnitine	MS/MS (+)	-0.750	-1.799	-9.401	-0.049	-1.067	-1.215	-3.528	-0.245	0.727
Hippuric acid	MS/MS (-)	-0.328	-0.931	-2.715	0.509	-0.418	-0.578	-3.146	0.182	0.407
Homoserine	GC-MS	-0.397	-0.381	-0.804	-0.164	-0.326	2.876	-1.093	49.272	0.727
Indole-3-acetyl-alanine	MS/MS (+)	-0.985	-1.268	-2.672	-0.191	-1.006	-1.097	-2.432	-0.158	0.727
Indoleacetyl glutamine	MS/MS (-)	-0.989	-2.223	-9.213	-0.153	-0.872	-1.030	-3.582	-0.262	0.315
Indoxyl sulfate	MS/MS (-)	-1.344	-1.627	-3.232	-0.379	-1.364	-1.710	-4.305	-0.005	0.896
Itaconic acid	GC-MS	-1.743	-1.645	-3.095	-0.681	-1.697	-1.620	-2.798	-0.856	0.793
Kynurenic acid	MS/MS (+)	-0.342	-0.337	-1.087	0.815	-0.227	-0.168	-0.974	1.586	0.458
Lactic acid	GC-MS	-1.323	-1.508	-3.924	-0.588	-0.845	-1.088	-3.596	4.349	0.295
Lactose	GC-MS	-0.206	-0.595	-2.471	0.252	-0.658	-2.467	-26.688	0.069	0.275
Leucine	GC-MS	0.751	0.837	-0.174	2.850	0.339	0.459	-0.547	1.801	0.138
Linoleamide	MS/MS (+)	0.808	0.672	-0.522	1.630	0.708	0.549	-1.028	1.423	0.458
Lysine	GC-MS	-0.055	-0.245	-5.344	1.561	-0.114	0.644	-0.338	5.255	0.727
LysoPC(18:2(9Z,12Z))	MS/MS (+)	-0.578	-2.150	-17.333	0.099	-0.675	-0.787	-2.686	0.132	0.458
LysoPC(22:2(13Z,16Z))	MS/MS (+)	0.395	0.289	-1.547	1.010	0.265	1.438	-3.796	15.299	0.793
M-cresol	GC-MS	-1.290	-1.376	-5.130	-0.029	-1.206	-1.590	-7.864	-0.007	0.965
Malonic acid	GC-MS	0.343	0.469	-1.004	1.979	0.945	0.792	-1.117	2.269	0.407
Mandelic acid	GC-MS	-0.925	-1.505	-4.831	-0.274	-0.820	-1.684	-8.469	-0.267	0.930
Mannitol	GC-MS	1.503	1.731	0.434	3.179	0.754	1.372	-0.379	6.076	0.061
Methylmalonic acid	GC-MS	-0.664	-0.783	-2.024	0.643	-1.148	-1.402	-3.529	-0.272	0.074
MG(0:0/16:0/0:0)	MS/MS (+)	0.474	0.500	-0.018	0.914	0.562	0.574	0.145	1.095	0.513
MG(18:0/0:0/0:0)	MS/MS (+)	0.485	0.475	-0.028	0.852	0.490	0.527	0.073	10.008	0.663
MG(18:2(9Z,12Z)/0:0/0:0)	MS/MS (+)	0.478	0.545	0.019	1.447	0.600	0.720	-0.137	1.547	0.383
Myristic acid	MS/MS (-)	0.491	0.322	-0.269	0.771	0.546	0.435	-1.068	1.624	0.407

Metabolite (T2-T0 Urine)	Method	Moderate Hypothermia (n=14) Median	Mean	Min	Max	Mild Hypothermia (n=15) Median	Mean	Min	Max	P value
N-acetyl-annosamine	GC-MS	-1.132	-1.007	-1.998	0.447	-1.087	-1.380	-3.926	0.353	0.337
Nonanoylcarnitine	MS/MS (+)	-1.580	-1.702	-4.216	-0.154	-1.315	-1.463	-3.952	-0.221	0.513
Octadecanamide	MS/MS (+)	0.262	-0.296	-6.475	0.687	0.246	-0.252	-6.125	0.918	0.793
Oxalacetic acid	GC-MS	-0.508	-0.676	-2.389	0.134	-1.200	-1.586	-4.939	0.381	**0.029**
Oxalic acid	GC-MS	0.380	0.880	-0.516	6.658	0.113	0.976	-1.178	11.769	0.513
Palmitoleic acid	MS/MS (-)	0.326	0.336	-0.704	1.340	0.372	0.445	-2.171	2.982	0.694
Phenylacetylglutamine	MS/MS (+)	-1.278	-1.480	-2.946	-0.105	-1.513	-1.348	-2.920	-0.089	0.896
Phenylethylamine	GC-MS	-1.787	-1.512	-2.650	0.041	-1.267	-1.105	-2.250	1.080	0.150
Phosphoric acid	GC-MS	-0.557	-0.729	-3.785	0.395	-0.299	-2.149	-22.842	0.098	0.827
Pipecolic acid	GC-MS	-0.163	-0.216	-0.929	1.204	-0.717	-0.551	-1.636	1.176	0.097
Ribitol	GC-MS	-0.047	1.289	-0.093	18.553	-0.082	2.532	-0.231	25.268	0.256
Ribose	GC-MS	0.291	1.077	-0.269	7.942	0.231	0.808	-0.927	4.206	0.315
S-adenosylhomocysteine	MS/MS (+)	-0.985	-1.838	-6.810	0.111	-1.893	-1.842	-4.389	0.065	0.513
Sebacic acid	MS/MS (-)	0.279	0.269	-0.896	1.061	0.290	0.242	-0.628	0.820	0.793
Serine	GC-MS	0.486	0.585	-0.243	2.210	0.281	0.512	-0.485	3.270	0.432
Sorbitol	GC-MS	-0.775	-0.947	-2.867	0.181	-0.986	-1.658	-5.264	-0.064	0.190
Sphingosine	MS/MS (+)	0.715	0.703	0.152	1.175	0.708	0.636	-0.114	1.058	0.760
Stearic acid	MS/MS (-)	0.459	0.576	-0.041	1.835	0.380	0.486	-1.112	1.561	0.930
Succinic acid	GC-MS	-0.651	-1.127	-3.256	-0.199	-1.123	-1.614	-5.659	-0.304	0.222
Threitol	GC-MS	-0.107	-0.549	-2.714	1.017	-0.118	-1.052	-7.723	0.999	0.485
Threonine	GC-MS	-1.014	-1.015	-1.615	-0.275	-1.241	-1.226	-2.066	-0.290	0.239
Ubiquinone-1	MS/MS (+)	-0.991	-1.324	-3.715	-0.230	-0.942	-1.250	-6.666	-0.035	0.631
Urea	GC-MS	-0.219	-0.175	-0.475	0.185	-0.182	-3.217	-19.688	0.191	0.827
Uric acid	MS/MS (-)	-0.554	-0.677	-1.691	-0.113	-0.730	-0.820	-2.111	-0.211	0.407
Xylitol	GC-MS	-0.529	-0.719	-1.935	-0.150	-0.580	-0.381	-2.568	2.721	0.759
α-Ketoglutaric acid	GC-MS	-0.586	-0.983	-4.581	0.339	-1.098	-1.821	-9.262	0.769	0.275

min: minimum, max: maximum, bold p-values indicate statistical significance. Cer: ceramide, MG: monoglyceride, LysoPC: lysophosphatidylcholine.
