# Supplementary material for: Differential metabolomic signatures in plasma and urine under mild and moderate hypothermia during cardiopulmonary bypass
Source: Sci Rep. 2025 Nov 20;15:41079. doi: 10.1038/s41598-025-24913-9 (PMC12634694; doi:10.1038/s41598-025-24913-9)
Supplement: Supplementary file 2 — Supplementary Material 2 [file 41598_2025_24913_MOESM2_ESM.pdf]

S.2 Material and Methods

S.2.1 Inclusion and Exclusion Criteria

Patients who required elective CPB surgery (coronary artery surgery, valve replacements and repairs, major artery surgeries), aged between 40 and 80, with a body mass index (BMI) of less than 35, stable kidney and liver functions, stable condition without respiratory support, no previous open-heart surgery, an ejection fraction (EF) greater than 40%, and those using histidine-tryptophan-ketoglutarate (HTK, Custodiol) cardioplegia solution were included in the study.

Patients with liver and kidney failure, low ejection fraction (less than 40%), use of cardioplegia solutions other than HTK, on respiratory or extracorporeal life support, those who received blood products (red blood cell suspension, plasma, etc.) during or before the operation, those with ongoing infections, those receiving immunosuppressive therapy, and those with tumors were excluded from the study based on exclusion criteria.

In addition, during metabolomic data preprocessing, three patients were excluded based on a predefined outlier criterion. Specifically, principal component analysis (PCA) was applied to both plasma and urine metabolomic datasets, and patients whose samples consistently deviated from the main data clusters in PCA score plots (outside the 95% confidence interval) were identified as outliers. These samples were removed prior to final statistical analysis to preserve data integrity and avoid bias (Figure S1.1).

The study was approved by the Ethics Committee of Ankara University Faculty of Medicine, and all participants provided written informed consent, as detailed in the main manuscript.

S.2.2 Sample Collection and Processing

Plasma samples were collected by using a 5 mL syringe to draw blood from a catheter placed in the right internal jugular vein immediately after anesthesia induction. To prevent potential contamination, the first 1 mL of blood (representing the catheter's internal fluid) was discarded. Then, 5 mL of blood was drawn and transferred into a purple-capped (EDTA) tube. These samples were centrifuged at 3100 rpm for 10 minutes, and the supernatant (plasma) was aliquoted into 1.5 mL Eppendorf tubes in 1 mL portions and stored at -80°C until metabolomic analysis.

Urine samples were collected from the reservoir of a urinary catheter placed after anesthesia induction into sterile urine containers, then aliquoted into 2 mL Eppendorf tubes and stored at -80 °C until metabolomic analysis.

S.2.3 Metabolomics Analysis

Plasma samples stored at -80 °C were thawed on ice, followed by adding 800 µL methanol:distilled water (9:1, v/v) and vortexed for 1 minute. The samples were then centrifuged at 15000 rpm for 10 minutes. From the supernatant, 250 µL aliquots were taken for both GC-MS and LC-qTOF-MS analyses and transferred into 1.5 mL Eppendorf tubes. Additionally, 100 µL from each sample was pooled to prepare quality control (QC) samples, and 250 µL aliquots from the QC pool were transferred into 1.5 mL Eppendorf tubes. All samples were dried in a vacuum centrifuge (Christ RVC 2-18 CD plus, Osterode am Harz, Germany). Urine samples stored at -80 °C were thawed on ice, followed by adding 100 µL urease (15 Units) and incubated at 37°C for 4 hours. Subsequently, 800 µL methanol/distilled water (9:1, v/v) was added and vortexed for 1 minute. Urine samples were prepared in the same manner as plasma samples and dried in a vacuum centrifuge along with QC samples.

S.2.3.1 GC-MS based metabolomics analysis

The dried samples were derivatized by adding 20 µL of methoxyamine hydrochloride solution, vortexed for 1 minute, and incubated at 30°C for 90 minutes. The samples were brought to room temperature and then 50 µL of N-methyl-N-trimethylsilyl trifluoroacetamide + trimethylchlorosilane (MSTFA + 1% TMCS) solution was added and vortexed for 1 minute, followed by incubation at 37°C for 30 minutes. The derivatized samples were analyzed under optimized conditions using a DB-5MS stationary phase column (30 m + 10 m duraguard x 0.25 mm i.d., 0.25 µm film thickness). The temperature program for the oven was configured as follows, with a total runtime of 37.5 minutes: initially maintained at 60 °C for 1 minute, followed by a ramp-up to 325 °C at a rate of 10 °C per minute, and then held at this temperature for 10 minutes. The injection volume was adjusted to 1 µL in splitless mode. Mass spectra were recorded for ions ranging from 50 to 650 m/z with an event time of 0.30 seconds. High-purity helium (>99.999%) was used as the carrier gas at a flow rate of 0.99 mL/min. The purge flow was set to 5 mL/min, and the injector temperature was maintained at 250 °C. The mass spectrometry (MS) parameters were specified as follows: the ion source temperature was set to

230 °C, the interface temperature was adjusted to 290 °C, and the solvent delay time was 5.91 minutes. Mass scanning was performed in the 50–650 m/z range with an event time of 0.30 seconds using GC–MS Solution (version 4.20). The derivatized samples were analyzed using a GC-MS system (GC-MS QP-2010 Ultra, Shimadzu, Japan) equipped with a DB-5MS column (30 m + 10 m DuraGuard × 0.25 mm i.d., 0.25 µm film thickness) under the optimized conditions specified in **Table S2.1.** Representative GC-MS chromatograms were given in the **Figure S2.2 A** and for plasma and **Figure S2.2** B for urine.

Table S2.1 GC-MS conditions for metabolomics analysis

PARAMETER	VALUE
Oven Temperature Program	Temperature increase from 60 °C (held for 1 minute) to 325 °C at 10 °C/min (held for 10 min)
Analysis Time	37.5 minutes
Injection Volume	1 µL (splitless)
Carrier Gas	Helium at 0.99 mL/min
Msd Transfer Line Temperature	290 °C
Solvent Delay Time	5.91 minutes
Mass Range	50-650 Dalton

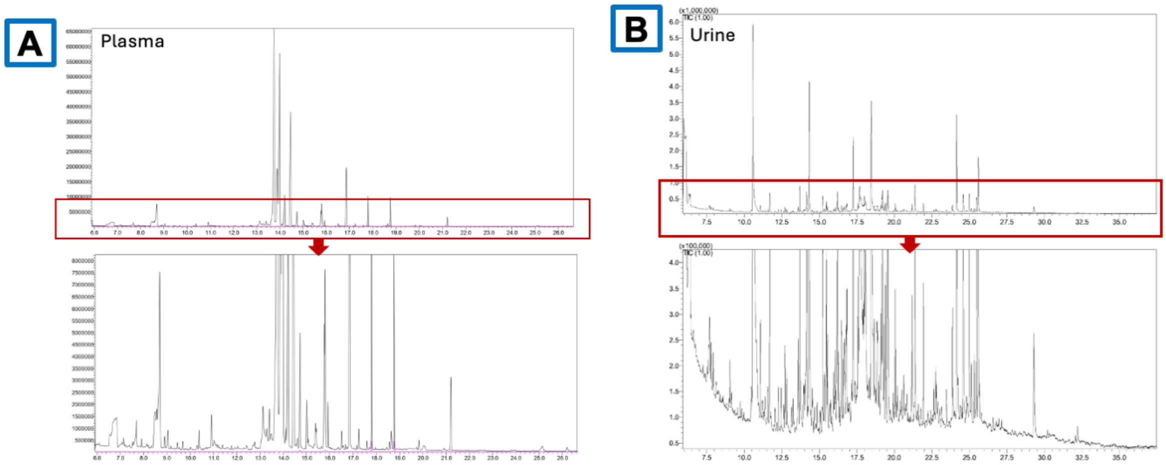

Figure S2.2 Representative GC-MS chromatograms; A) Plasma samples B) Urine samples

S.2.3.2 LC-qTOF-MS based metabolomics analysis

For LC-qTOF-MS based metabolomic profiling, dried samples were reconstituted in 200 µL of 0.1% formic acid (FA) in acetonitrile and water, vortexed for 1 minute, and centrifuged at 10,000 rpm for 10 minutes. A total of 175 µL of supernatant was transferred into vials and injected into the LC-qTOF-MS system (Agilent 6530, Agilent Technologies, USA).

Chromatographic separation was performed using a C18 column (1.0 × 150 mm, 2.7 µm) under gradient elution conditions, as detailed in **Table S2.3**. The mobile phases consisted of (A) water containing 0.1% formic acid and (B) acetonitrile containing 0.1% formic acid. The flow rate was maintained at 0.15 mL/min. The gradient initiated with 10% mobile phase B, maintained for 1 minute, then progressively increased to 90% by 1-14 minutes and was held between 14-15 minutes. This was followed by a decrease from 90% to 10% by 20 minutes and maintained at 10% until 25 minutes to allow for column re-equilibration. The column temperature was controlled at 60°C throughout the procedure. QC samples, prepared by pooling all individual samples, were analyzed with different collision energies (10, 20, and 40 eV) in targeted MS/MS mode to facilitate peak identification. The total ion chromatograms (TIC) from the LC-qTOF-MS analysis are presented in **Figure S2.5**, showing representative chromatograms for plasma samples (positive and negative ionization; **Figure S2.5A**) and urine samples (positive and negative ionization; **Figure S2.5B**). The qTOF-MS parameters included the following settings: gas temperature at 300°C; nebulizer pressure at 35 psi; drying gas flow rate at 8 L/min; VCap voltage at 3500 V; fragmenter voltage at 175 V; and skimmer voltage at 65 V. Further instrument details are provided in **Table S2.4**.

Table S2.3 Gradient elution program

TİME (MİN)	% MOBİLE PHASE B
0	10
1	10
14	90
15	90
20	10
25	10

*% 0.1 FA İN ACETONİTRİLE

Table S2.4 LC-qTOF-MS instrument parameters.

Parameter	Positive Ionization	Negative Ionization
Mass range (amu)	50-650	50-650
Scan speed (spectrum/s)	2	2
Spray voltage (kV)	3500	3500
Skimmer voltage (V)	65	65
Gas temperature (°C)	300	300
Gas flow rate (L/min)	8	8
Nebulizer (psi)	35	35

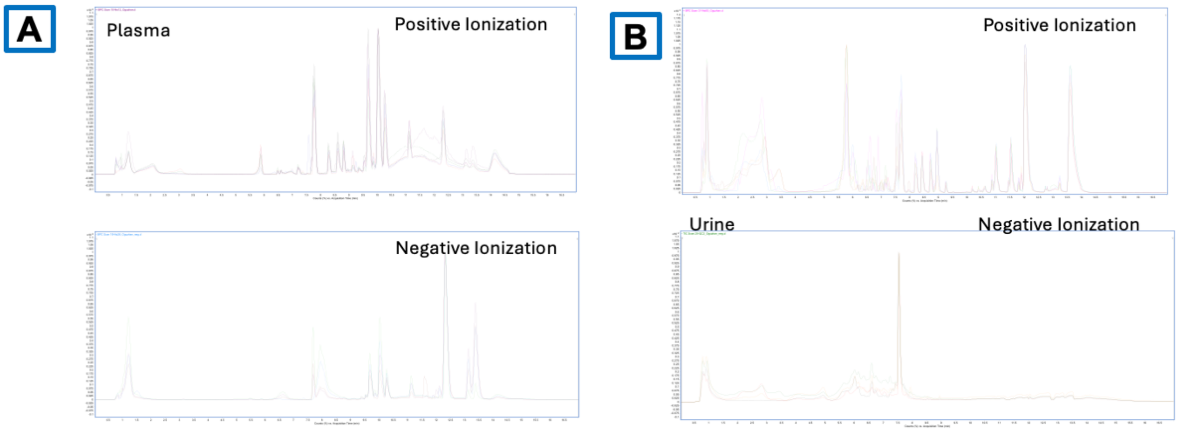

Figure S2.5 Representative LC-qTOF-MS chromatograms; A) Plasma samples (pozitive and negative ionization) B) Urine samples (pozitive and negative ionization)

S.2.4 Metabolomic Data Analysis and Statistical Analysis

Complex chromatograms from GC-MS and LC-qTOF-MS based metabolomic analyses were processed using MS-DIAL (ver. 4.92) software to deconvolute, align retention times, and generate data matrices. The data were normalized according to total peak area of the total ion chromatogram. Metabolite peaks from GC-MS analyses were identified using retention index-based Fiehn and Golm libraries, and the data matrices were exported to Excel. Peaks in the data matrices from LC-qTOF-MS analyses were identified by comparing MS/MS spectra from QC samples, obtained at different energy levels (10, 20, and 40 eV), with open-access spectral libraries (positive and negative, RIKEN) provided by the software manufacturer.

Given the data's complexity, multivariate statistical techniques were used for evaluation. After data curation, data matrices from urine and plasma samples were combined and transferred to SIMCA14.1 software for analysis using Principal Component Analysis (PCA) and Partial Least Squares Discriminant Analysis (PLS-DA). These analyses identified significant metabolites and regression coefficients crucial for group differentiation. PCA plots were created to visualize any systematic errors or outliers in the dataset, while PLS-DA plots were used to visualize the differentiation between all data groups and pairwise comparisons. This allowed for detailed

data interpretation and identification of key metabolites responsible for group differences. Variable importance in projection (VIP) plots from PLS-DA analyses helped identify the significantly altered metabolites. Coefficient plots were used to determine which metabolites increased or decreased concentration within specific groups. The reliability of PLS-DA models was assessed using R^2 and Q^2 values.

Statistical analyses in this study were conducted using R software (R Core Team, 2024). The analyses utilized onewaytests (Dag et al., 2018) and stats (R Core Team, 2024) packages. The Wilcoxon test was applied to compare the time points at which the samples were collected (T_1-T_0, T_2-T_0), allowing for the identification of statistically significant metabolites. Following this step, Mann-Whitney U test was used to compare two independent groups with respect to the change between the compared time points. , Mann-Whitney U test was also employed to compare independent groups in terms of time independent continuous variables.

For descriptive statistics, measures such as median, mean, minimum- maximum values were reported. Additionally, for categorical data analysis, Pearson's chi-square test and Fisher's exact test were used when comparing two independent groups. Contingency tables were generated, and Fisher's exact test was applied if the expected value in any cell was below 5; otherwise, Pearson's chi-square test was used. Results were reported based on frequency and percentage values. A significance threshold of 0.05 was adopted for statistical evaluations. P-value < 0.05 indicated a statistically significant relationship, whereas $p > 0.05$ implied no significant association.

Supplementary File S2.5: Cardiopulmonary Bypass Circuit and Equipment Details

To ensure standardization across all patients, a uniform cardiopulmonary bypass (CPB) circuit setup was used. The tubing set configuration consisted of a 3/8-3/32 inch PVC arterial line, a 1/2-3/32 inch silicone arterial pump head, a 1/2-3/32 inch PVC venous line, and 1/4-1/16 inch PVC tubing for the sucker and vents. The oxygenator used in all cases was the Trillium Affinity model, manufactured by Medtronic (USA). The heart-lung machines employed during CPB procedures included the Stockert S5 (LivaNova, Germany) and Quantum (Spectrum Medical,

United Kingdom). Occlusion and calibration adjustments were made regularly in the heart-lung machines.s

The prime solution for CPB was Isolyte-S, with a total volume of 1600 cc. The CPB circuit was primed with 1600 cc of Isolyte-S before extracorporeal circulation was initiated. Arterial line filtration and bubble trap mechanisms were utilized to ensure optimal perfusion safety. Perfusion flow rates and pressure management were adjusted according to institutional perfusion protocols.

S2.6. Anesthesia Protocol and Timing

All patients underwent a standardized anesthesia protocol. Anesthesia was induced with intravenous fentanyl and propofol. Prior to cardiopulmonary bypass (CPB), maintenance anesthesia included sevoflurane (inhalation) and remifentanil (infusion). During CPB, remifentanil and propofol infusions were continued. After CPB, anesthesia was maintained with remifentanil infusion and sevoflurane. Minor adjustments were made according to individual clinical status. Based on routine surgical workflow in our clinic, the average time between anesthesia induction and T0 sampling was approximately 45–55 minutes.

REFERENCES

Eylem, C. C., Nemutlu, E., Dogan, A., Acik, V., Matyar, S., Gezercan, Y., Yildirim, E., Turkez, H., & Akduman, N. E. B. (2025). Optimized high-throughput protocols for comprehensive metabolomic and lipidomic profiling of brain sample. Talanta, 282, 126953.

Dag, O., Dolgun, A., Konar, N. M. 2018. "onewaytests: An R Package for One-Way Tests in Independent Groups Designs", The R Journal, 10(1), 175-199.

R Core Team (2024). R: A language and environment for statistical computing. R Foundation for Statistical Computing, Vienna, Austria. URL https://www.R-project.org/.
